# Development and Characterization of Natamycin-Loaded Liposomes for Potential Topical Application: Influence of Preparation Method and Phospholipid Composition

**DOI:** 10.3390/ph19050710

**Published:** 2026-04-30

**Authors:** Natalija Čutović, Petar Batinić, Tatjana Marković, Andrea Pirković, Ninoslav Mitić, Jovana Petrović, Aleksandra A. Jovanović

**Affiliations:** 1 Institute for Medicinal Plants Research “Dr. Josif Pančić”, Tadeuša Košćuška 1, 11000 Belgrade, Serbia; pbatinic@mocbilja.rs (P.B.); tmarkovic@mocbilja.rs (T.M.); 2 Institute for the Application of Nuclear Energy INEP, University of Belgrade, Banatska 31b, 11080 Belgrade, Serbia; andrea.pirkovic@inep.co.rs (A.P.); ninoslavm@inep.co.rs (N.M.); ajovanovic@inep.co.rs (A.A.J.); 3 Department of Plant Physiology, Institute for Biological Research “Siniša Stanković”—National Institute of Republic of Serbia, University of Belgrade, Bulevar Despota Stefana 142, 11000 Belgrade, Serbia; jovana0303@ibiss.bg.ac.rs

**Keywords:** skin application, controlled release, nanoparticle tracking analysis, antibiofilm activity, natamycin, liposomal vesicles

## Abstract

**Background/Objectives**: Natamycin is an effective antifungal limited by poor solubility. This study aimed to develop and characterize natamycin-loaded liposomal vesicles as a biocompatible delivery system to improve stability and achieve controlled release for potential topical application in the treatment of fungal infections. **Methods**: Formulations were prepared using two phospholipid mixtures (Lipoid S100 and Phospholipon 90H) *via* standard (thin-film) and proliposome methods. Evaluation included encapsulation efficiency (EE%), particle size, zeta potential, the polydispersity index (PDI), and rheological properties. *In vitro* release kinetics were compared to a natamycin solution. Antifungal efficacy was tested against four *Candida* strains to determine minimum inhibitory and fungicidal concentrations (MICs and MFCs, respectively) and biofilm inhibition, while biocompatibility was assessed *via* keratinocyte viability assays. **Results**: Formulations achieved high encapsulation (~90%). Natamycin incorporation improved homogeneity and reduced particle diameters, particularly in proliposome-derived vesicles, suggesting strong drug–lipid interactions. Preparation method and lipid type significantly influenced properties; thin-film formulations showed a lower PDI and higher stability. Diffusion was twofold slower than the control, with Lipoid S100 proliposomes providing the most sustained release. The liposomes demonstrated robust antifungal activity (MICs: 0.00625–0.2 mg/mL) and effective biofilm inhibition against *C. krusei*. While high concentrations moderately reduced keratinocyte viability, lower doses remained biocompatible and slightly stimulatory. **Conclusions**: Lipid composition and preparation methods have minimal impact on the physical properties and *in vitro* release profiles of natamycin liposomes. These vesicles provide a dose-dependent, biocompatible platform for the controlled delivery of antifungals, showing significant *in vitro* inhibitory activity against *Candida* growth and biofilm formation.

## 1. Introduction

Natamycin, also known as pimaricin or tennectin [1], is a natural antimycotic polyene that is characterized by the presence of a large macrocyclic lactone ring, which contains several conjugated double bonds, as well as one or more sugar residues [2]. This antifungal agent is naturally produced by *Streptomyces natalensis* and is characterized by a molecular weight of 665.7 Da [3]. The use of natamycin as an antifungal agent is widely known, especially in the food industry and medicine [4,5]. It has a highly specific mode of action, as it kills yeasts by binding to the ergosterol present in their structure, although without permeabilizing the membrane of the plasma, thus inhibiting vacuolar fusion [6]. As a result, natamycin is highly effective against various yeasts and molds, but ineffective against bacteria and viruses. But, as Ture et al. [6] have previously reported, natamycin, like some other active components, may have a sudden drop in active concentration due to rapid diffusion from the solution. In addition to this, it is widely known that natamycin is characterized by its low stability and solubility in water (approximately 30–50 mg/mL) [7,8,9]. The encapsulation of active components into suitable carriers leads to a decrease in the rate of their diffusion, thus aiding the maintenance of a favorable concentration at the intended location of their application, while also prolonging their overall stability and bioavailability of the encapsulated compounds [10,11].

Liposomal vesicles are spherical lipid-based vesicles that have long been employed for targeted drug delivery [12,13]. A wide range of bioactive compounds (water-soluble, lipid-soluble, and amphiphilic compounds) has recently sparked growing interest in applications within both functional foods and non-food sectors, such as pharmaceuticals and cosmetics, owing to their biocompatibility, biodegradability, and targeting capabilities [14,15]. The feasibility of industrial-scale liposome production, combined with their valuable feature of targetability, offers significant advantages for pharmaceutical applications. In recent years, new scalable manufacturing protocols for liposome production have been developed, including the thin-film method [16], the proliposome method [11,17], and a heating-based method [18]. Furthermore, the use of natural components in liposome formulation, ensuring biocompatibility, biodegradability, and non-toxicity, facilitates their incorporation into dermo-cosmetic systems while easing regulatory constraints [19]. The physical properties of the bilayers, including rigidity, fluidity, and surface charge, are largely determined by their specific lipid composition [20]. The thin-film hydration method is among the most commonly applied techniques for liposome preparation. It involves forming a thin lipid layer on the inner surface of a rotary evaporator flask after solvent removal [11,19]. The resulting lipid film is subsequently hydrated using water or an appropriate buffer solution. Prior to hydration, both the lipid film and the aqueous phase are typically heated above the lipid phase transition temperature (T_m_), when required, to facilitate proper bilayer formation [16]. Vigorous agitation, often combined with sonication in an ultrasonic bath, assists the detachment of the lipid film from the flask surface and promotes liposome formation. Liposomal vesicles produced by this method are mostly multilamellar vesicles (MLVs) with heterogeneous size distribution. However, the thin-film technique has several limitations, including relatively low encapsulation efficiency for hydrophilic compounds, broad particle size distribution that necessitates additional size-reduction steps, and the use of organic solvents that must be thoroughly removed [21]. Furthermore, the procedure is relatively time-consuming and not easily scalable for large-scale production.

The proliposome method represents a comparatively simple approach for liposome preparation. Unlike the thin-film method, its main drawback is lower reproducibility when small batches are prepared, although it often provides higher encapsulation efficiency. In this technique, lipids are first dissolved in a mixture of water and ethanol while being stirred at approximately 60 °C for about 10 min to form a homogeneous lipid paste [20,22]. The mixture is then cooled, after which water or buffer is gradually added dropwise under continuous stirring. The resulting suspension is allowed to hydrate for approximately one hour, during which MLVs are formed.

Liposomal vesicles are amongst the most widely used forms of encapsulation for various biologically active compounds, as they are compatible with the encapsulation of hydrophilic and hydrophobic compounds [23]. Besides this, they show high biodegradability and compatibility with human skin cells, making them favorable for use in the pharmaceutical industry, thus being a good choice for the encapsulation of natamycin performed in this study [24].

Encapsulation of active compounds into liposomal particles has been a focus of a wide variety of research, as it improves their stability, solubility, compatibility, and controlled release into the preferred medium [19,20,22,25]. Natamycin, however, exhibits very low water solubility and is characterized by fast diffusion into the bulk, which limits its prolonged use, making the development of an appropriate encapsulation method highly valuable. Therefore, this study aims to evaluate the effect of two phospholipid mixtures, as well as two liposomal preparation methods chosen based on the research group’s expertise in developing skin-compatible formulations, on the obtained liposomal particles with respect to the efficiency of the encapsulation process and the prolonged release, antimicrobial, antibiofilm, and cytotoxic potential of natamycin encapsulated in the liposome carrier. The liposomal particles were further characterized using nanoparticle tracking analysis (NTA) and transmission electron microscopy (TEM) to determine their size distribution and stability, while photon correlation spectroscopy (PCS) was used to measure the zeta potential and polydispersity index (PDI) of developed vesicles. In addition, density, surface tension, and viscosity were measured to provide practical insights for their potential application in the pharmaceutical industry, in the form of gels, creams, or lotions. To the best of the authors’ knowledge, this is the first study to demonstrate the encapsulation of natamycin using both the proliposome and thin film methods for the development and *in vitro* characterization of liposomal formulations as a basis for potential topical delivery systems.

## 2. Results

In the present study, natamycin-loaded liposomal vesicles were prepared using thin-film and proliposome methods and subsequently characterized in terms of their EE%, their structure, size distribution, concentration, heterogeneity, and surface charge (using TEM NTA, and PCS analyses, respectively), as well as density, viscosity, and surface tension. Their biological effects, including antifungal and antibiofilm potential, and cytotoxicity towards HaCaT cells, were also evaluated. Additionally, the diffusion resistance, total mass transfer, and *in vitro* release kinetics for all of the natamycin-loaded liposomal vesicles were determined using the Franz diffusion cell in simulated physiological conditions.

### 2.1. Encapsulation Efficiency

**Figure 1:**
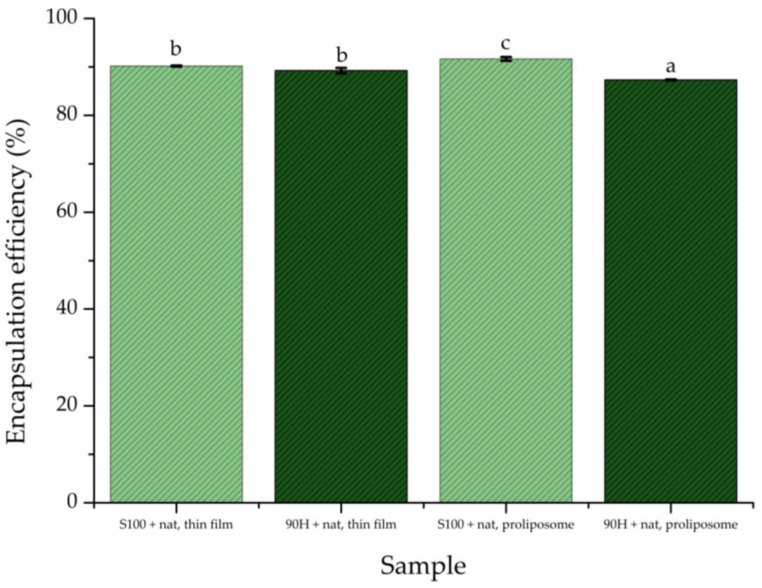
Encapsulation efficiency of developed liposomal formulations with natamycin (N) using thin film (tf) and proliposome (pl) techniques and Lipoid S100 and Phospholipon 90H phospholipids; different letters show a statistically significant difference (*p* < 0.05; n = 3, one-way ANOVA, Tukey’s *post hoc* test); data are presented as mean ± standard deviation.

EE% represents the most important criterion for the determination of the ability of the liposomal systems to act as carriers for different types of biologically active compounds, either in their lipid bilayers or aqueous core [26]. The EE% of the natamycin-loaded liposomal vesicles is determined right after their preparation, and the results are presented in Figure 1.

The highest EE% was achieved when natamycin was encapsulated into liposomal vesicles prepared using Lipoid S100 as the phospholipid mixture and the proliposome method, reaching approximately 91.64%. Conversely, the lowest amount of natamycin was encapsulated into liposomal vesicles prepared *via* the same proliposome method but using Phospholipon 90H (~87.31%). The liposomal vesicles prepared using Lipoid S100 as the phospholipid mixture showed a significantly higher EE% than the ones prepared using Phospholipon 90H-containing liposomal vesicles in both employed liposome preparation techniques. Precisely, the Lipoid S100 liposomal vesicles achieved an EE% of over 91%, whereas the ones with Phospholipon 90H were below 90%. This difference may be attributed to the distinct phase transition temperatures (T_m_) of phospholipids: the unsaturated Lipoid S100 has a significantly lower T_m_ than the saturated Phospholipon 90H, resulting in more fluid bilayer membranes that facilitate the incorporation of the encapsulated natamycin [27]. In the literature, it can be observed that molecules most similar to natamycin in terms of chemical structure and reactivity, such as nystatin (characterized by a macrolide lactone ring and an associated chain containing conjugated double bonds) [28], demonstrate a high degree of encapsulation in liposomes, reaching approximately 80% [29]. Furthermore, in this study, liposomes were prepared using unsaturated phospholipids, such as Lipoid S100, which, due to its composition and high purity, significantly reduces membrane rigidity and increases bilayer fluidity. Similar results were also obtained in a study by Moribe et al. [30] that employed unsaturated phospholipids to prepare liposomes encapsulating nystatin. On the other hand, as all of the liposomal formulations contain cholesterol (a member of the sterol group), it can be assumed that interactions between cholesterol and Lipoid S100 are stronger. Its ability to strengthen the bilayer and reduce membrane permeability may synergistically contribute to the higher EE% [24,31]. The research conducted by Gelen-Gungor et al. [32] shows an EE% of ~94.2% for nisin and ~86.4% for azithromycin when both are prepared using the thin film method, which is in line with our findings. On the other hand, in the case of propolis (as an antimicrobial agent) encapsulated into liposomal vesicles, using the proliposome method, the EE% was ~60% [33], depending on the formulation, thus showing that the level of encapsulation of natamycin achieved by this method is high.

From the perspective of the influence of the preparation method on liposome formation and the EE% of natamycin, subtle differences were observed depending on both lipid composition and preparation technique. The thin-film hydration method yielded comparable EE% values for formulations based on S100 and Phospholipon 90H, suggesting that lipid saturation has a limited effect under this preparation approach. In contrast, the proliposome method exhibited a more pronounced dependence on lipid composition, with the S100-based formulation achieving the highest EE%, while the 90H system showed the lowest value. Therefore, the results indicate that unsaturated phospholipids, i.e., Lipoid S100, when combined with the proliposome method, tend to enhance the encapsulation efficiency of natamycin, whereas saturated phospholipids, Phospholipon 90H, are associated with reduced EE%, particularly in proliposome-based systems [11].

It should be noted that the quantification of natamycin was performed using a UV–Vis spectrophotometric method that was not fully validated according to standard analytical validation guidelines (e.g., accuracy, precision, robustness, and detailed matrix effect evaluation were not assessed). Although the method demonstrated acceptable linearity within the tested range and no significant interference from blank liposomal formulations, potential limitations related to specificity in complex lipid matrices and possible interference from degradation products cannot be entirely excluded. The applied method was used as a rapid and practical approach for comparative purposes (screening of formulations), where all samples were analyzed under identical conditions. Therefore, the results obtained are considered reliable for relative comparison of EE% and *in vitro* release profiles. Nevertheless, for more rigorous quantitative analysis, particularly in complex delivery systems, the use of more selective analytical techniques such as high-performance liquid chromatography (HPLC) would be recommended.

### 2.2. Transmission Electron Microscopy Data of Developed Liposomal Vesicles with Natamycin

**Figure 2:**
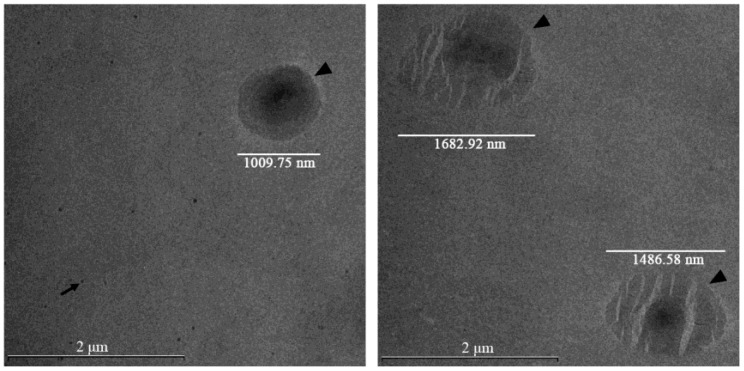
Representative transmission electron microscopy images of natamycin-loaded liposomal vesicles containing Phospholipon 90H and produced by using the thin film method; bar—2 µm. The arrow points to the natamycin particles, whereas the arrowhead shows the liposomal vesicle.

TEM was employed to visualize the size, homogeneity, integrity, and morphological characteristics of liposome preparations. Representative TEM images of Phospholipon 90H/proliposome-based natamycin-loaded liposomal vesicles are presented in Figure 2. Additionally, TEM images of the remaining developed natamycin-loaded liposomal vesicles are presented in Figure S1.

In Figure 2, the left panel shows intact liposomal vesicles containing Phospholipon 90H and natamycin with a structure typical of successful thin film hydration preparation and associated with high EE% and sustained drug release. In contrast, the right panel depicts ruptured vesicles containing Phospholipon 90H and natamycin, which arise due to stress during drying and solvent removal, resulting in disrupted membranes, potential drug leakage, and altered release profiles. The comparison between these micrographs highlights the critical role of processing conditions in determining liposome stability and functional performance. TEM images are presented as representative morphological evidence and are not intended for quantitative size analysis.

The TEM images presented in Figure S1 (Supplementary Materials) show liposomal vesicles loaded with natamycin prepared using the proliposome method and Phospholipon 90H (A), the proliposome method and Lipoid S100 (B), and the thin film method and Lipoid S100 (C). Differences in morphology, size, and lamellarity can be observed depending on the method used and the type of phospholipids. However, independent of the phospholipid composition, liposomal vesicles prepared using the proliposome method displayed irregular morphology, lack of sphericity, and poorly defined boundaries, which may reflect insufficient vesicle stabilization and possible membrane disruption (Figure S1A,B). Additionally, irregular liposome morphology may affect formulation stability, drug encapsulation efficiency, and controlled release of natamycin. Furthermore, uniform, spherical vesicles are preferred for predictable skin penetration and consistent topical delivery. Notably, liposomal vesicles prepared by the proliposome technique using the hydrogenated phospholipid Phospholipon 90H showed visible natamycin aggregates in TEM micrographs as well (Figure S1A). This formulation also demonstrated the lowest EE% (Figure 1), suggesting limited affinity of natamycin for the rigid hydrogenated lipid matrix and insufficient entrapment within the vesicular structure. Such morphological irregularities, including aggregation and poorly formed vesicles, are commonly associated with suboptimal rehydration and inadequate bilayer self-assembly during proliposome preparation, potentially leading to reduced drug retention and physical instability [34]. Conversely, the thin-film hydration method produced well-formed, uniformly spherical liposomal vesicles with sharp and well-defined edges (Figure 2 and Figure S1C).

The observed morphological differences between developed liposome formulations can be attributed to the inherent characteristics of the preparation techniques and the physicochemical behavior of the lipids under processing conditions. Specifically, the proliposome method often produces liposomal dispersions with higher heterogeneity and irregular structures due to incomplete or uncontrolled vesicle rehydration, which can lead to drug crystallization or aggregation outside the lipid bilayer [23,26]. In contrast, the thin-film hydration method promotes more uniform bilayer self-assembly during solvent removal and hydration, contributing to improved structural integrity and more homogeneous liposome populations [35]. These differences are consistent with previous reports that highlight the influence of preparation method on the morphology, size distribution, and encapsulation performance of liposomal drug carriers, where method selection plays a critical role in determining vesicle quality and functional behavior in application [11,36]. 

One limitation of the present study is that liposome morphology was assessed using conventional TEM, which may induce vesicle deformation or partial collapse due to dehydration during sample preparation. Although the obtained images confirmed the presence of vesicular structures, this technique may not fully reflect the native morphology of liposomal vesicles in the hydrated state. Future studies should therefore employ Cryo-TEM, which allows visualization of liposomal vesicles under near-native conditions and may provide more detailed insight into vesicle structure and drug localization.

### 2.3. Results of Nanoparticle Tracking Analysis and Photon Correlation Spectroscopy of Developed Liposomal Vesicles

The NTA provided detailed insights into the size distribution and particle concentration of plain and natamycin-loaded liposomal vesicles. Differences in phospholipid composition (Phospholipon 90H vs. Lipoid S100) and preparation approach (thin film vs. proliposome) were reflected in distinct size profiles and particle concentrations, allowing for comparative evaluation of formulation efficiency and stability.

**Figure 3:**
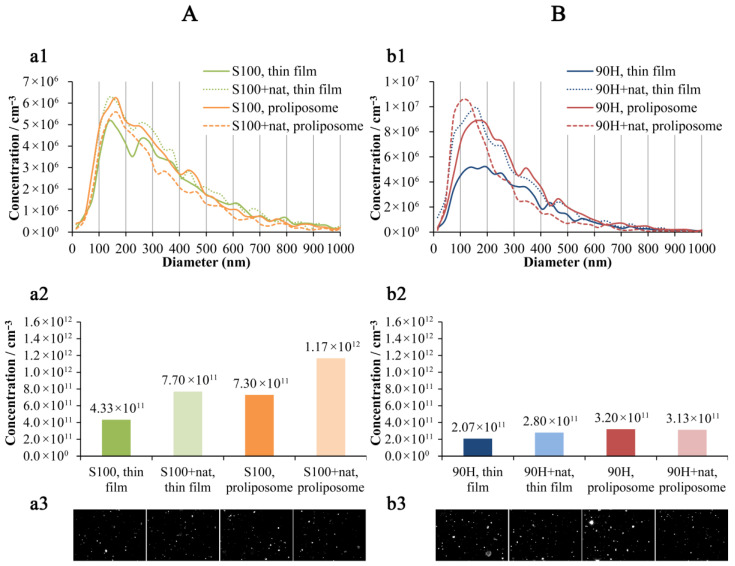
Characterization of plain and natamycin-loaded liposomal vesicles (prepared using thin film or proliposome techniques) using nanoparticle tracking analysis (NTA): (**A**) Lipoid S100 liposomal vesicles (size distribution (**a1**), concentration (**a2**), and representative video frame capture (**a3**), respectively) and (**B**) Phospholipon 90H liposomal vesicles ((size distribution (**b1**), concentration (**b2**), and representative video frame capture (**b3**), respectively)), showing the particles in each preparation; nat, natamycin.

As can be seen in Figure 3A, natamycin-loaded Lipoid S100 liposomal vesicles showed a lower median diameter (279 nm and 252 nm) in comparison to the plain parallels (295 nm and 267 nm) in both employed preparation techniques (thin film and proliposome methods, respectively). Additionally, the liposomal vesicles prepared using the thin film method possessed a higher median diameter compared to the counterparts obtained in the proliposome method. Consequently, the liposome concentration was higher in the formulations with natamycin (7.70 × 10^11^ particles/cm^−3^ and 1.17 × 10^12^ particles/cm^−3^) vs. unloaded liposomal vesicles (4.33 × 10^11^ particles/cm^−3^ and 7.30 × 10^11^ particles/cm^−3^), while the poliposome method provided the samples with a higher concentration of liposomal particles than the thin film method in both plain and loaded liposomal vesicles. The obtained phenomenon was expected since the same amount of phospholipids was used in all the liposome preparations, while the addition of natamycin and conditions in the proliposome method provided the smaller diameter particles. The same trend related to the median diameter can be observed in the case of Phospholipon 90H liposomal vesicles (Figure 3B). Namely, plain Phospholipon 90H liposomal vesicles had a median diameter of 247 nm and 237 nm, while the liposomal vesicles with encapsulated natamycin possessed a median diameter of 212 nm and 168 nm. The Phospholipon 90H liposomal vesicles from the thin film technique showed a higher diameter than those from the proliposome method as well. However, the concentration of liposomal particles was higher in the presence of natamycin (2.80 × 10^11^ particles/cm^−3^) vs. empty liposomal vesicles (2.07 × 10^11^ particles/cm^−3^) only in the case of the liposomal vesicles prepared employing the thin film method. On the other hand, the Phospholipon 90H samples (plain and loaded liposomal vesicles) obtained in the poliposome method showed a similar concentration of liposomal particles (~3.20 × 10^11^ particles/cm^−3^). This outcome may reflect differences in lipid composition and phase transition behavior between Lipoid S100 and Phospholipon 90H phospholipids, influencing vesicle size, stability, and formation efficiency [37,38]. The decrease in particle size upon drug loading may be attributed to interactions between natamycin and the phospholipid bilayer, which can enhance membrane packing density and reduce vesicle curvature [39]. A decrease in detected liposome size after natamycin loading has been previously observed and attributed to structural rearrangements of the lipid bilayer upon incorporation of the drugs (such as natamycin and others), leading to tighter supramolecular packing and altered bilayer organization [40,41]. Such intercalation effects have also been shown to influence the zeta potential, polydispersity index, and bilayer packing in drug-loaded lipidic systems. The zeta potential of the same liposomal formulations, in our publication, was previously reported to be approximately −25 mV, indicating good colloidal stability over a 60-day storage period [11]. In addition, liposomal vesicles prepared *via* the thin-film method consistently displayed a higher median diameter than those obtained using the proliposome technique, which is in line with previous findings indicating that the proliposome method provides more uniform vesicle formation and smaller particle sizes due to improved hydration dynamics and shear conditions during dispersion [42,43,44]. Consequently, the concentration of liposomal particles was higher in natamycin-loaded samples compared to unloaded liposomal vesicles, particularly for the proliposome-derived formulations. This inverse relationship between particle size and particle count is expected, given the fixed phospholipid quantity used in all preparations. Namely, smaller liposomal vesicles correlate with a higher number of particles at constant lipid content [45]. The observed size reduction and enhanced particle concentration upon natamycin incorporation suggest favorable drug–lipid interactions and the efficiency of the proliposome technique for producing smaller, more concentrated liposomal systems. All Lipid S100 liposomal vesicles possessed a higher average particle diameter and concentration (Figure 3A) in comparison to the Phospholipon 90H parallels (Figure 3B). The larger vesicle size of Lipoid S100 systems can be attributed to compositional differences between the two phospholipids. Lipoid S100, being rich in unsaturated phosphatidylcholine species, tends to form more fluid bilayers with reduced packing density, which facilitates vesicle fusion and growth during hydration and extrusion [46,47]. In contrast, the higher hydrogenation degree of 90H results in a more ordered and rigid bilayer structure, promoting the formation of smaller and more stable vesicles [47]. The higher particle concentration observed for Lipoid S100 formulations may arise from the enhanced dispersion of lipid fragments during film hydration or from incomplete aggregation suppression in softer bilayer systems. Lower concentration in the Phospholipon 90H liposomal formulations, despite lower vesicle size, can be explained by the production of foam during liposome preparation and consequently loss of lipids. These findings align with previous studies reporting that lipid composition, particularly the degree of acyl chain saturation and transition temperature, plays a pivotal role in controlling vesicle size, lamellarity, and particle count in liposomal dispersions [46,47].

In the present study, the zeta potential and PDI of the developed liposomal formulations (empty and natamycin-loaded) were also determined as key physicochemical parameters (using the PCS method). These measurements are of fundamental importance for a more comprehensive characterization of liposomes, as they provide insight into particle stability, surface charge, and size distribution. Evaluating these properties enables a better understanding of the behavior and quality of the liposomal system. The results are presented in the Supplementary Materials (Table S1). The PDI and zeta potential results indicate that both phospholipid type and preparation method significantly influence liposome characteristics. Formulations prepared by the thin film method exhibited lower PDI values, suggesting a more uniform size distribution compared to proliposomal systems, which showed higher heterogeneity, particularly in 90H-based formulations. S100 liposomes demonstrated lower PDI values (except for plain proliposomes), indicating better homogeneity. The incorporation of natamycin reduced PDI in formulations, suggesting a stabilizing effect. The zeta potential values of all formulations were negative, indicating the presence of electrostatic repulsion between vesicles and suggesting acceptable colloidal stability. Thin film liposomes based on 90H exhibited the most negative values (~−41.00 mV for blank and ~−37.98 mV with natamycin), indicating higher stability compared to other systems. In contrast, S100 thin film liposomes showed fewer negative values (~−28.54 mV for blank and ~−32.90 mV with natamycin), suggesting slightly lower electrostatic stabilization. The incorporation of natamycin led to a small increase (related to the absolute value) in negative charge in S100 formulations, while a slight decrease or the absence of the change was observed in 90H systems. Specifically, in S100 proliposomes, the blank formulation showed a more negative value (~−35.32 mV) compared to the natamycin-loaded one (~−30.23 mV), suggesting that drug incorporation may reduce surface charge in this system. Namely, 90H-based thin film liposomes demonstrated the highest electrostatic stability, while proliposomal formulations, particularly those based on S100, showed greater variability, indicating that both lipid type and preparation method influence surface charge properties.

While the particle size, polydispersity index, and zeta potential of developed liposomal formulations were monitored over a 2-month period in our previous study [11], a leakage study and temperature-dependent stability were not assessed in the present work and will be addressed in future studies to provide a more comprehensive evaluation of liposomal stability.

### 2.4. Rheological Properties of Liposomal Vesicles

The measurements of density, surface tension, and viscosity provided valuable information on how different phospholipids and the employed preparation techniques, as well as the incorporation of natamycin, affect the physical properties of the liposomal systems (Table 1).

Density differences, even if small, can affect suspension stability and may correlate with internal structure or entrapped volume [48]. As shown in Table 1, the density of Phospholipon 90H liposomal vesicles was ~0.997–1.001 g/mL, while Lipoid S100 liposomal vesicles had slightly higher values (~1.007 g/mL), independent of natamycin loading. This indicates that the preparation method had little influence on density, while lipid composition contributed to minor differences, possibly due to chain length, unsaturation, headgroup packing, or lipid fraction per unit volume (also supported by NTA, Section 2.3, Figure 3A). Near-unity density for all samples confirms that these formulations are largely aqueous. The literature shows that liposome density can vary with cargo loading and bilayer composition [48].

Surface tension measurements showed more pronounced variation (Table 1), reflecting vesicle interfacial behavior and potential bilayer/surfactant interactions [20,45]. Phospholipon 90H liposomal vesicles exhibited higher surface tension than Lipoid S100 in the thin-film method, whereas the proliposome method produced similar values for both lipids. Natamycin loading slightly increased surface tension in all cases, suggesting minor disruption of the interfacial layer and bilayer packing. Lower surface tension in Lipoid S100 systems indicates greater interfacial activity, likely due to lipid composition. These results highlight the influence of lipid type and preparation method on interfacial properties, which may impact suspension stability and vesicle size distribution.

Viscosity of all formulations was low (~1.6–2.3 mPa·s) but increased upon natamycin loading, with the largest rise in Phospholipon 90H proliposome liposomal vesicles (from 1.76 to 2.26 mPa·s), suggesting higher internal content, drug loading, or inter-vesicle interactions. Lipoid S100 formulations showed smaller increases. Viscosity trends align with particle size, lamellarity, and interparticle interactions [48,49,50,51]. The increase in viscosity upon drug incorporation may also reflect partial bilayer reorganization, as natamycin intercalates between lipid acyl chains, enhancing packing and reducing mobility [49,51]. Higher viscosity in proliposome Phospholipon 90H systems may indicate multilamellarity, consistent with its influence on flow behavior [52,53]. Although the liposomal suspensions exhibit low viscosity, they are intended to be incorporated into topical pharmaceutical formulations such as creams, lotions, gels, ointments, sprays, or adhesive hydrogel patches, ensuring suitable application, controlled delivery, and potentially enhanced skin absorption of natamycin.

The rheological properties of the developed formulations are of particular importance for their application behavior. The observed flow behavior suggests that the formulations can be easily spread under shear stress during application, while retaining sufficient viscosity at rest to ensure prolonged residence time at the site of application. Such characteristics may be relevant for further development of topical delivery systems, as they contribute to improved applicability, and enhanced skin retention.

### 2.5. In Vitro Release-Based Comparative Study

**Figure 4:**
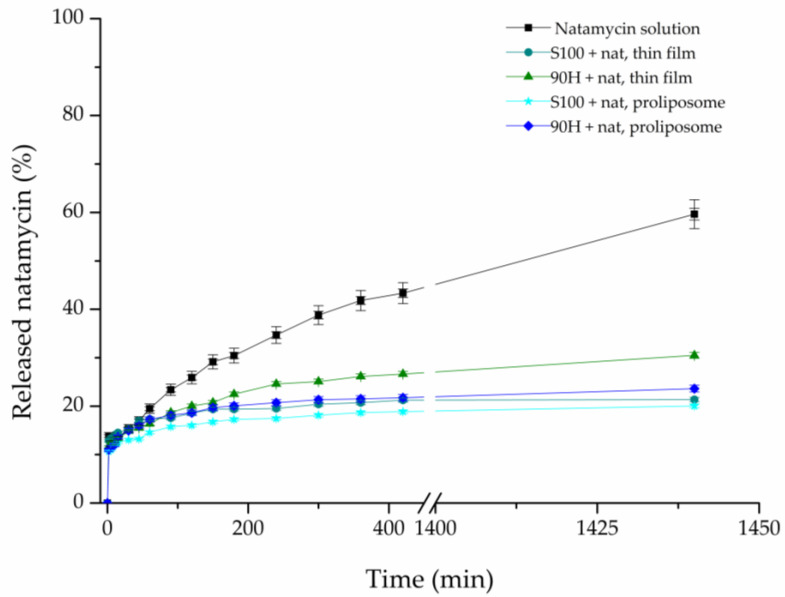
Release profiles of natamycin from the natamycin (nat) solution and liposomal vesicles prepared by thin-film and proliposome methods using Lipoid S100 and Phospholipon 90H phospholipids, expressed as the percentage of natamycin released in phosphate-buffered saline at pH ~ 5.5 and 37 °C. Data are presented as mean ± standard deviation (n = 3).

The comparative release studies of natamycin from liposomal vesicles were performed using a Franz diffusion cell to quantify the resistance of the liposomal membrane to mass transfer. The results are shown in Figure 4, showing the percentage of released natamycin over 24 h in a PBS medium (pH ~5.5) as a function of time. The *in vitro* release of natamycin from all four natamycin-loaded liposome formulations was compared with the diffusion profile of a pure natamycin solution containing the same concentration of the drug as in the liposomal vesicles. 

As presented in Figure 4, the diffusion of natamycin from its solution happened very fast, at almost twice the rate as from the liposomal vesicles, in the same medium, which was to be expected. After a period of 24 h had passed, the amount of released natamycin from its solution was approximately 59.64%, whereas in the case of liposomal vesicles, it ranged from ~20.05% to ~30.5%. These results show that liposomal vesicles can be used for the prolonged release of natamycin, thus aiding the future development of products with this antifungal agent.

When discussing the influence of the liposome preparation method on release properties, our previous study demonstrated the advantages of the proliposome method compared to the conventional Bangham technique for the preparation of liposomes [11]. In line with these findings, the literature suggests that thin-film hydration typically yields heterogeneous, often multilamellar vesicles that may exhibit a pronounced initial burst release due to structural non-uniformity. In contrast, proliposome-derived systems form more stable and uniform liposomes upon hydration, resulting in a more controlled and sustained release profile [54,55].

**Figure 5:**
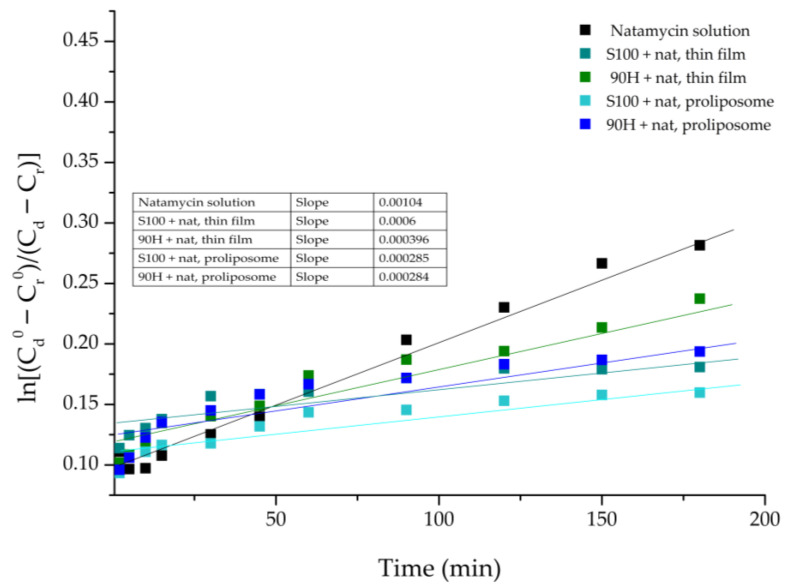
Dimensionless plot of natamycin concentration as a function of time for the release curves from the natamycin (nat) solution and liposomal vesicles prepared by thin film and proliposome methods using Lipoid S100 and Phospholipon 90H phospholipids, expressed as the percentage of natamycin released in phosphate-buffered saline at pH ~ 5.5 and 37 °C.

The *in vitro* release study data were further analysed to calculate the diffusion coefficient (D) and the diffusion resistance (R) associated with the liposomal bilayers in the aqueous system. These results are shown in Table 2. The coefficient for natamycin diffusion from its solution, as well as natamycin-loaded liposomal vesicles, was calculated from the slope of the linear part of the curve defined by plotting the function: $\ln\left(\frac{C_{d}^{0}-C_{r}^{0}}{C_{d}-C_{r}}\right)$ vs. time, t (Figure 5).

The value of R represents the cumulative resistance of the used semipermeable acetate-cellulose membrane, as well as the resistance that the liposomal bilayer provides. The resistance to the diffusion of natamycin that is detected in the case of the pure natamycin solution originates from the acetate-cellulose membrane used in the experiment. Keeping this in mind, the resistance to diffusion from the liposomal vesicles can be determined by subtracting the membrane resistance from the total resistance. 

From the parameters of diffusion, which are shown in Table 2, it can clearly be seen that there is an evident difference in the diffusion of natamycin from its solution and liposomal formulations. The highest resistance to the diffusion of natamycin was calculated in the case of Lipoid S100-based liposomal vesicles prepared using the proliposome method. Namely, it was to be expected, as the release rate was the slowest in the case of this liposomal formulation. On the other hand, the lowest diffusion resistance was in the case of the natamycin solution, due to the fact that the natamycin molecules must only withstand the resistance of the synthetic membrane and can otherwise move unrestrictedly. This difference in the diffusion resistance can be explained by the fact that, in the case of natamycin-loaded liposomal vesicles, natamycin must first start to release from the liposomal particles, by breaking the physical interactions with the phospholipids, followed by the diffusion through the membrane [20]. From the perspective of physical interactions, it is important to emphasize that hydrophobic interactions with the lipid bilayer describe the affinity of nonpolar regions of a molecule for the hydrophobic core of membrane phospholipids. In the case of natamycin, its polyene macrolide structure enables strong surface-level association with lipid acyl chains, leading to membrane adsorption and interaction with sterols without deep bilayer penetration [56]. Additionally, the differences that occurred in the diffusion resistance between the liposomal formulations prepared using various phospholipid mixtures can be explained by the distinct chemical makeup of the mixtures, as Lipoid S100 is made up of unsaturated and saturated fatty acids, whereas Phospholipon 90H contains saturated fatty acids [52]. The distinct chemical compositions of the two phospholipids lead to variations in EE%, as shown in Section 2.1. *Encapsulation Efficiency*, owing to the differing rigidity of the resulting membranes, which in turn affects the release behavior [11,53]. Furthermore, this highlights the capacity of phospholipid membranes to regulate molecular transport through enhanced structural rigidity and diminished permeability when different phospholipid mixtures are used [57]. Interestingly, the lower diffusion resistance calculated in the case of both liposomal formulations prepared using Phospholipon 90H can also be attributed to the higher temperatures that were needed to transform the lipid mixture into the liquid state in the first step, as well as for hydration to be efficient, that could have led to the degradation of the membrane, and thus causing leakage, and easier release of the drug [58]. In comparison to our previous work [11], the release of natamycin at a pH~5.5 is almost two times slower than it was at a pH~7.2. These findings indicate that liposomal particles can be used to control the release of natamycin at the skin pH (~5.5), while also showing which lipid mixture provides greater drug retention.

The release of natamycin from all prepared liposomal formulations was subjected to kinetic analysis, using three different models. Specifically, Higuchi, First-order, and Korsmeyer-Peppas kinetic models were used, and the obtained results are presented in Figures S2–S6 and Tables S1–S3. The majority of the obtained release profiles for natamycin from all tested formulations showed high consistency with all three models, with the exception of the natamycin-loaded liposomal vesicles obtained using Lipoid S100 and the proliposome method (R^2^ < 0.900). The release of natamycin according to the Higuchi model points to the dominant influence of molecular diffusion, while the linearity that occurs between the cumulative release and the square root of time points to Fickian diffusion. A high consistency with the First-order model points to a fast release of the drug from the liposomal vesicles at the beginning of the diffusion, and slowing down after some time, which is in accordance with the results obtained from the experiment.

Also, the main criterion used to assess whether the applied model was accurate was the obtained R^2^ value (Table S1). Namely, the closer the R^2^ value is to 1, the better the kinetic model fits for predicting values [59]. According to the results of this study, the majority of the analyzed liposomal formulations met this specific criterion. Therefore, both of the used methods for liposomal preparation (proliposome and thin film) produced liposomal particles that showed a prolonged release profile, in comparison to the natamycin solution, thus pointing to their potential for use in the pharmaceutical industry.

Natamycin exhibits low aqueous solubility (30–100 mg/L at room temperature), which is expected to increase at physiological temperature (37 °C). Under the applied experimental conditions, partial sink conditions were likely maintained during the initial and intermediate stages of the release study; however, complete sink conditions cannot be assumed throughout the entire experiment. Therefore, the release data should be interpreted in a comparative rather than an absolute manner.

The present study provides a preliminary *in vitro* evaluation of liposomal formulations, focusing on drug release behavior under controlled conditions, while skin permeation and retention studies remain necessary for further confirmation of topical applicability. Namely, a limitation of the current study is the absence of *ex vivo* skin permeation and retention experiments, which are critical for directly confirming the liposomal formulation’s targeting and penetration capabilities. Future studies will incorporate these experiments to provide a comprehensive evaluation of liposome-skin interactions and validate the potential of the system for topical delivery of natamycin.

### 2.6. Biological Activities of Natamycin-Loaded Liposomal Vesicles

#### 2.6.1. Antimicrobial Activity of Natamycin-Loaded Liposomal Vesicles

**Figure 6:**
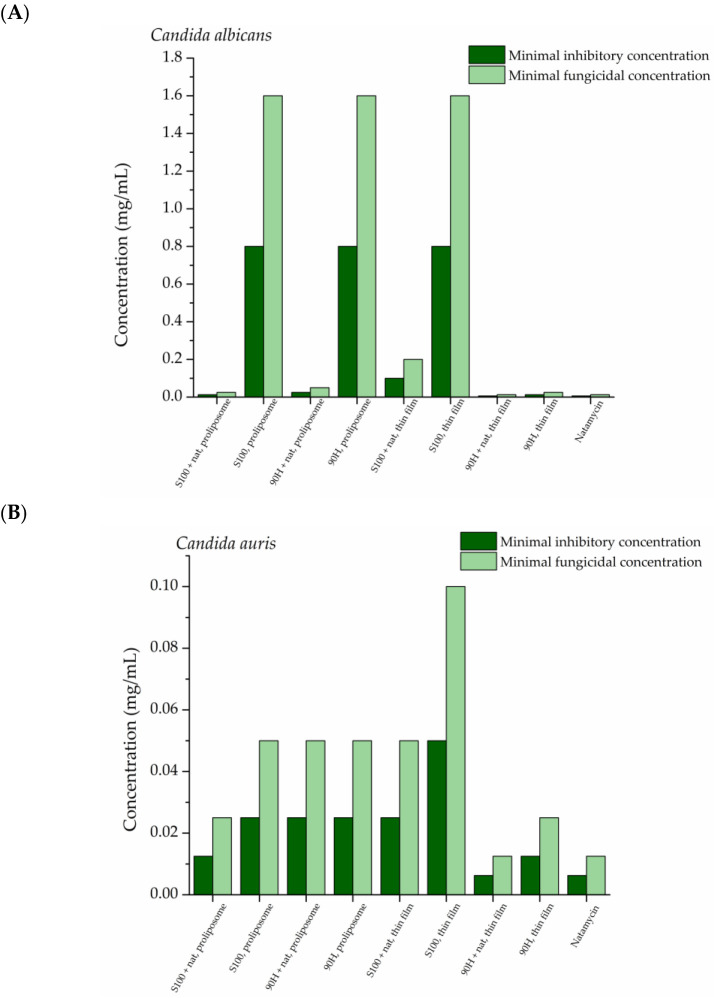

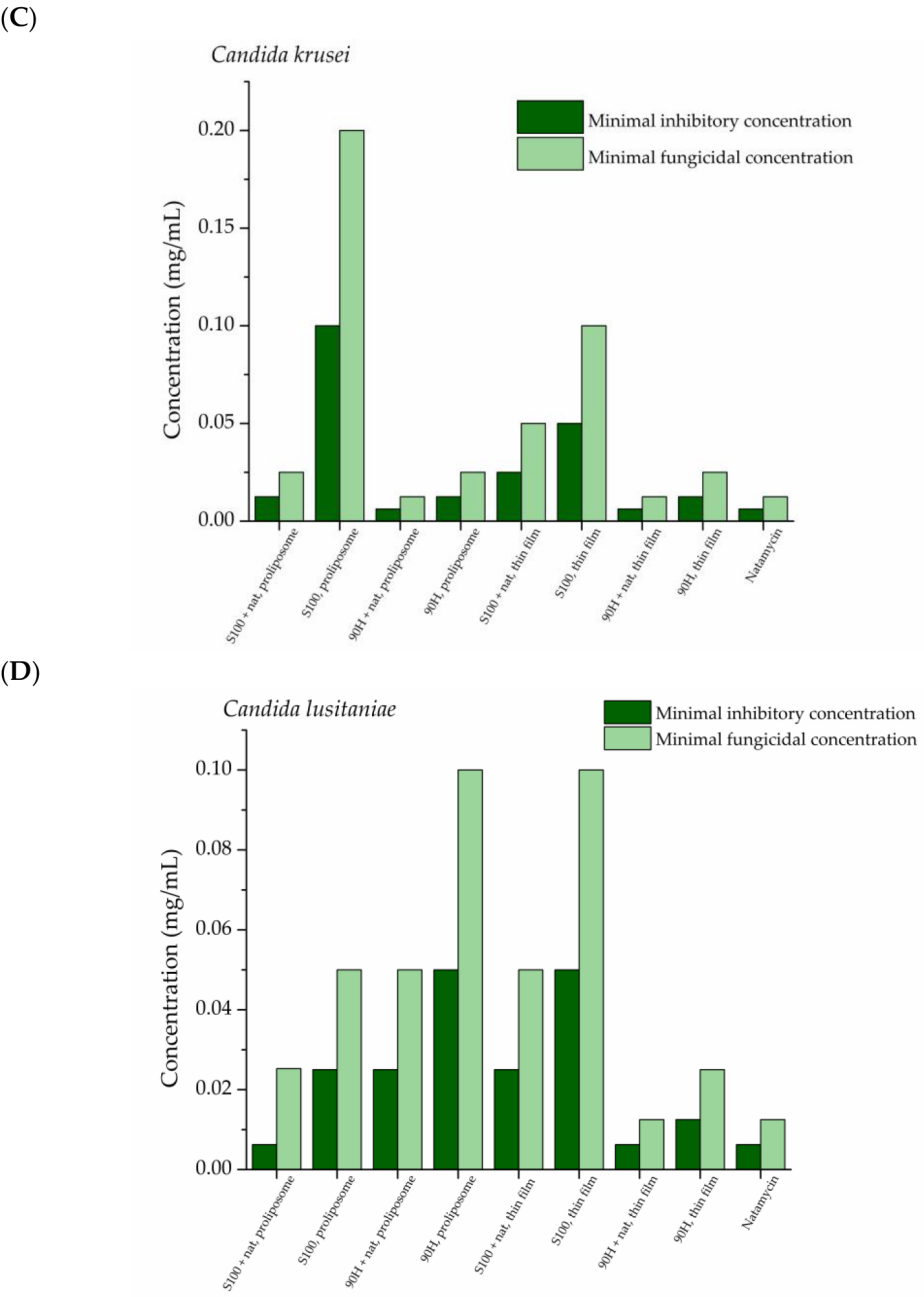
Antifungal activity of natamycin (nat), blank (control), and natamycin-loaded liposomal vesicles prepared using thin-film and proliposome techniques, and Lipoid S100 and Phospholipon 90H phospholipids against (**A**) *Candida albicans*, (**B**) *Candida auris*, (**C**) *Candida krusei*, and (**D**) *Candida lusitaniae*, expressed as minimal inhibitory and minimal fungicidal concentrations (MIC and MFC, respectively); “stricter criteria” rule was used, as is usual in antimicrobial tests.

The liposomal vesicles with encapsulated antifungal agent, natamycin, were subjected to the testing of their activity in inhibiting the growth of four *Candida* species: *C. albicans*, *C. lusitaniae*, *C. auris*, and *C. krusei* (Figure 6).

The difference in the antimicrobial potential of all liposomal formulations against the four tested *Candida* strains is clearly presented in Figure 6, as well as the difference in comparison to the natamycin solution. All of the natamycin-loaded liposomal vesicles are efficient in inhibiting the growth of the four tested fungal strains in a dose-dependent manner, as the MIC ranged from 0.00625 mg/mL to 0.2 mg/mL, while the MFC values were in the range from 0.0125 mg/mL to 0.4 mg/mL, depending on the *Candida* strain, as well as the liposomal formulation. The *Candida* species that has shown to be the least susceptible to the influence of natamycin from liposomal vesicles was *C. albicans*, for which the MIC was from 0.0125 mg/mL to 0.2 mg/mL. On the other hand, the natamycin solution used as the positive control was equally efficient against all of the tested fungal strains, i.e., the MIC values were 0.00625 mg/mL, while the MFC ones were 0.0125 mg/mL.

Antifungal activity of the liposomal formulations was evaluated in comparison with the free natamycin solution at the same drug concentration, allowing assessment of whether liposomal encapsulation influences antifungal potency. The obtained MIC and MFC values, therefore, provide a direct comparison between the encapsulated and non-encapsulated antifungal agents. The occurrence that the MIC and MFC values were higher in the case of encapsulated natamycin than when it is in its free form can be explained by the slower release of natamycin from liposomes than from the solution. After a 24 h period, ~60% of natamycin is released from the solution, whereas in the case of natamycin-loaded liposomes, it reached a maximum of ~30% (90H, thin film). This confirms that the encapsulation process preserves the antifungal activity of natamycin while enabling controlled release from the lipid carrier.

The liposomal formulation that had the lowest MIC values in the case of three pathogens (*C. krusei*, *C. lustiniae*, and *C. auris*) was the one prepared using Phospholipon 90H and the thin film method, but it was the least efficient in the case of *C. albicans*. The occurrence mentioned about the three strains can be explained by the fast release of natamycin from the liposomal bilayer (Section 2.5. *In Vitro Release-Based Comparative Study*), which leads to the presence of higher concentrations of the antifungal agent over a shorter period of time. This is in accordance with previous findings that liposomal vesicles can be used as carriers for various antimicrobial agents, due to their ability to mimic the membrane of the pathogens, allowing for easier release [11]. Furthermore, the phenomena that in the case of *C. auris* the very same liposomal formulation showed the highest MIC and MFC values can be brought into connection with the incompatibility of the fungal cell walls and the used lipid mixture, which could have led to reduced penetration of natamycin into the cell wall, as the MIC is also high in the case of the control (blank) parallel (0.8 mg/mL) [60].

Natamycin is an antifungal agent with a long tradition of being used in the pharmaceutical, food, and cosmetic industries, in order to treat various fungal infections or prolong the product’s shelf life [61]. In the current era of increasing antimicrobial resistance, it is especially important to assess the effect of antimicrobial agents in their encapsulated form to see whether it affects the drug-resistant pathogens. As it is known, natamycin has been widely used to treat eye infections caused by fungi from the *Fusarium*, *Aspergillus*, and *Candida* spp. [62], but has not been investigated thoroughly against *Candida* strains usually found on human skin.

Previous studies that have assessed the encapsulation of natamycin into nanoparticles [63,64,65] have shown that its entrapment into suitable carriers leads to the improvement of its controlled release, as well as penetration into the ocular and skin cells [64]. With this in mind, it is to be expected that the MIC values are relatively low due to the synergistic effects of both the phospholipid mixture and natamycin [65]. To the best of the authors’ knowledge, there have not been previously published studies about the effects of natamycin-loaded liposomal vesicles against skin fungi.

#### 2.6.2. Antibiofilm Potential of Liposomal Vesicles

**Figure 7:**
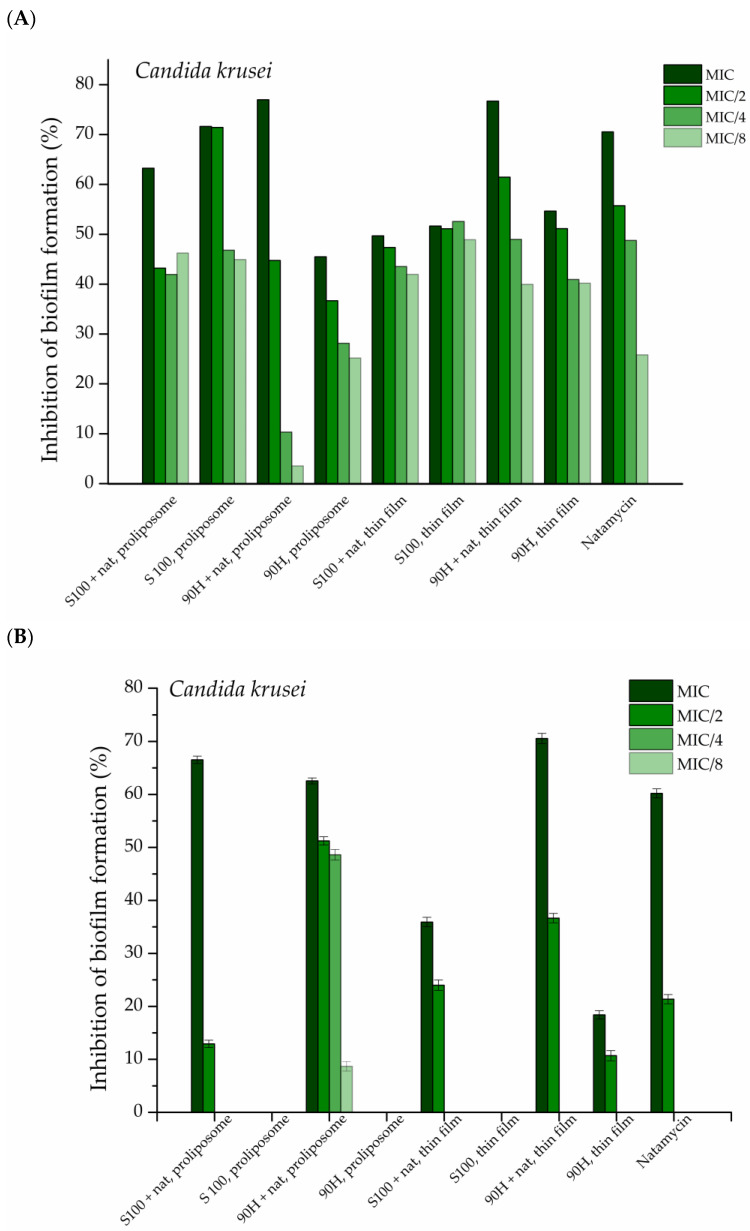
Inhibition of biofilm formation (%) for (**A**) Candida krusei and (**B**) Candida lusitaniae, determined by the CV assay; results are presented as mean value (%) ± standard deviation (SD). MIC—minimal inhibitory concentration. (The antibiofilm potential was assessed for pure natamycin (nat) all natamycin-loaded and blank liposomal vesicles, while the missing bars point to no activity in inhibiting biofilm formation).

*Candida lusitaniae* and *C. krusei* were included in this study because they are clinically relevant non-albicans *Candida* species with increasing incidence in the general population, as well as resistance to conventional antifungals. This allows assessment of the natamycin-loaded liposomal formulation against pathogens with limited susceptibility to commonly used antifungals. The tested natamycin solution and natamycin-loaded liposomal vesicles concentrations were the MIC determined in the antifungal assay, their half value (MIC/2), quarter value (MIC/4), and one-eighth of the value (MIC/8). The obtained results are presented in Figure 7.

Natamycin activity showed dependence on both liposome type and preparation method, although some results showed inconsistency not strictly attributable to any of these factors. Overall, Phospholipon 90H-based liposomal vesicles exhibited better activity than Lipoid S100-based ones obtained by both the thin film and proliposome methods in the case of both tested *Candida* strains. For *C. krusei*, the distinction between formulations was less clear, as control values were in several cases comparable to liposomal vesicles loaded with natamycin (Figure 7A). In the case of *C. lusitaniae*, all natamycin-loaded liposomal vesicles performed better than the control (which showed no inhibition, therefore was not added in Figure 7B), with Phospholipon 90H-based liposomal vesicles prepared by the thin film method exerting the best antifungal effect. The natamycin-loaded liposomal vesicles prepared using Lipoid S100, with both methods for liposomal preparation, showed inhibition at the tested concentrations but remained less effective than the Phospholipon 90H-based liposomal vesicles manufactured by the thin film method. Even so, Phospholipon 90H-based liposomal vesicles, particularly the ones obtained by the thin film technique, displayed higher inhibition than Lipoid S100 in both methods, whereas the proliposomal formulations showed greater variability and occasional overlap with controls.

In summary, Phospholipon 90H-based liposomal vesicles, particularly those prepared using the thin film method, demonstrated the most consistent improvement of natamycin activity in inhibiting biofilm formation for both tested *Candida* species, whereas the proliposome formulations showed greater variability and, in some cases, values comparable to the controls.

The antifungal agent, natamycin, has previously been shown to be efficient in inhibiting the growth of various *Candida* species and their biofilms [15,66,67]. The results for the inhibition of fungal biofilm formation are in accordance with the ones acquired for the antifungal potential of the liposomal formulations (the ones obtained using Phospholipon 90H as the lipid mixture, even though both lipid mixtures originate from soybean); Phospholipon 90H is composed of pure hydrogenated phospholipids, whereas Lipoid S100 is made up of phosphatidylcholine [20]. To the best of the authors’ knowledge, there is no previous work performed on the effects of natamycin-loaded liposomal vesicles on either *C. krusei* or *C. lusitaniae* biofilm formation.

#### 2.6.3. Cytotoxicity of Developed Liposomal Vesicles

**Figure 8:**
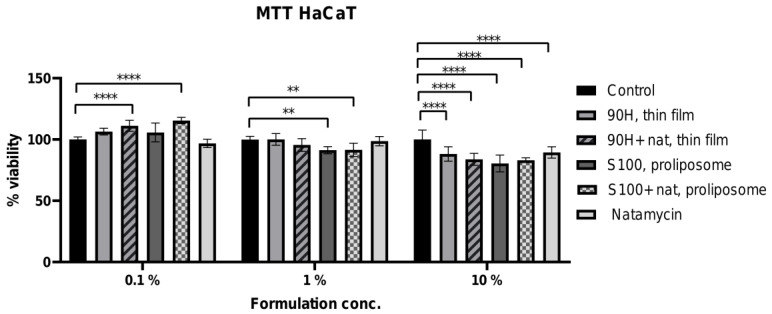
Cytotoxicity of pure natamycin (nat), plain and natamycin-loaded liposomal vesicles prepared using the thin-film technique and Phospholipon 90H or proliposome technique and Lipoid S100, in a range of concentrations (0.1, 1, and 10% of liposomal formulation, i.e., 1, 10, and 100 µg/mL of natamycin, respectively), determined by MTT assay in HaCaT cells. Data are expressed as mean + SD (standard deviation) relative to the unexposed control; ** *p* < 0.01, **** *p* < 0.0001 by one-way analysis of variance (ANOVA) with Tukey’s multiple comparison *post hoc* test.

The cytotoxic potential of the two selected liposomal formulations (Lipoid 100S liposomal vesicles obtained using the proliposome method and Phospholipon 90H liposomal vesicles obtained using the thin film method, due to the highest antimicrobial effect) was evaluated using the HaCaT cell line as a model of human epithelial non-cancerous cells. This assessment provides essential information on the biocompatibility and safety profile of the formulations for potential biomedical or pharmaceutical applications. *In vitro* cell models are widely used in cytotoxicity testing for reliable, reproducible, and cost-effective screening of new compounds, reducing the reliance on animal testing while assessing crucial mechanisms behind the efficacy and safety of chemicals, pharmaceuticals, cosmetics, nanoparticles, and biological agents [68]. The viability of cells following treatment was determined using the MTT assay, allowing quantitative comparison between pure natamycin, plain, and natamycin-loaded liposomal vesicles. The data are presented graphically in Figure 8.

As expected, the highest concentration of natamycin and liposomal vesicles with encapsulated natamycin resulted in a significant reduction in the HaCaT cell viability across all tested formulations, demonstrating a clear cytotoxic effect (Figure 8). The negative control (non-treated cells) is included in the experiment and is represented by the black bars in Figure 8. Cell viability in the negative control was 100 ± 7.68%, and the standard deviations of the treated groups (10% formulation) were comparable, indicating that the observed variability is within the range of the control group. Statistically significant differences (**** *p* < 0.0001) were observed at the 10% formulation concentration, while remaining consistent with the variability of the negative control. Specifically, at the highest tested concentration (10% of liposomal sample, i.e., 100 µg/mL of natamycin in loaded liposomal vesicles or solution), all liposomal formulations, with or without natamycin, as well as free natamycin, reduced cell viability in HaCaT cells, indicating a concentration-dependent inhibitory effect on cellular metabolic activity. The observed decrease in cell viability at a higher concentration may be attributed to increased lipid-cell membrane interactions and higher local drug exposure, which can transiently affect membrane integrity and mitochondrial activity measured by the MTT assay. Namely, a moderate decrease in cell viability was detected, particularly for the Lipoid S100/proliposome-based formulations, yet all values remained above 80%. The slightly higher cytotoxicity of Lipoid S100/proliposome-based liposomal vesicles compared to Phospholipon 90H/thin film liposomal vesicles is likely attributable to the phospholipid composition. Lipoid S100 is a highly purified phosphatidylcholine (~≥94%) derived from natural soybean phospholipids, with a relatively high content of unsaturated fatty acids in the lipid chains (e.g., linoleic and oleic acids). The double bonds in the unsaturated fatty acids are more prone to oxidation, particularly under formulation, storage, or processing conditions. Lipid oxidation can generate reactive aldehydes or peroxides, which may compromise liposomal membrane integrity and increase cytotoxicity when applied to cells [69]. Phospholipon 90H, being hydrogenated (~90% phosphatidylcholine), contains mostly saturated fatty acids. Saturated lipids are more chemically stable and less susceptible to oxidation [70], which likely contributes to the lower cytotoxicity observed for proliposome-based liposomal vesicles. The higher cytotoxicity of Lipoid S100-based thin film liposomal vesicles could be due to the oxidation of the unsaturated fatty acids, producing reactive species that interact with keratinocytes, whereas the hydrogenated Phospholipon 90H provides a more stable lipid bilayer and better biocompatibility. Notably, free natamycin exhibited slightly higher cell viability compared to the encapsulated forms at this concentration (100 µg/mL). These results suggest that careful dose selection is important to maintain biocompatibility while achieving therapeutic efficacy. The MIC values of developed liposomal vesicles (expressed as the natamycin concentration, presented in Section 2.6.1. *Antimicrobial Activity of Natamycin-Loaded Liposomal Vesicles*) ranged from 6.25 µg/mL to 200 µg/mL. Although some MIC values overlap with the higher tested concentrations, the moderate decrease in viability observed even at 100 µg/mL indicates an acceptable balance between antifungal efficacy and cellular compatibility for topical use. Several studies have reported that blank liposomal formulations exhibit low or negligible cytotoxicity toward HaCaT cells, indicating good biocompatibility of the lipid carriers. For example, liposomal vesicles at appropriate concentrations were found to be non-cytotoxic in HaCaT cells using the MTT assay, while the observed toxicity was mainly attributed to the encapsulated active compound rather than the liposomal vehicle itself [71,72]. Natamycin is considered a safe antifungal agent, and advanced delivery systems such as natamycin-loaded lipid nanoparticles or derivatives have shown improved therapeutic performance without significant cytotoxic effects on ocular or epithelial tissues [73,74]. Further, lower concentrations (0.1% and 1% of liposomal vesicles, i.e., 1 and 10 µg/mL of natamycin in loaded liposomal vesicles, respectively) showed minimal impact, indicating that the formulations are potentially biocompatible at therapeutic levels. At the intermediate concentration (1% of liposomal vesicles), only the proliposome-based formulations containing Phospholipon 90H (in the absence and presence of natamycin) caused a slight reduction in cell viability, while thin film-based liposomal vesicles containing Lipoid 100S and free natamycin showed no noticeable negative impact on cell survival. In contrast, at the lowest tested concentration (0.1% of liposomal vesicles), both natamycin-loaded liposomal systems (Lipoid S100/thin film liposomal vesicles and Phospholipoid 90H/proliposome liposomal vesicles) slightly increased the viability of HaCaT cells compared to the control, suggesting not only biocompatibility but also a potential stimulatory effect on keratinocyte metabolic activity at low doses. Thus, these results demonstrate a clear concentration-dependent response and confirm the acceptable cytocompatibility of the developed liposomal formulations, making them suitable for potential topical applications.

In summary, the *in vitro* study demonstrated a clear concentration-dependent effect on HaCaT cell viability, with all liposomal formulations showing acceptable cytocompatibility at lower concentrations and moderate, formulation-dependent reductions at higher concentrations, suggesting their potential suitability for further investigation in topical delivery application.

## 3. Materials and Methods

### 3.1. Standards and Reagents

**Figure 9:**
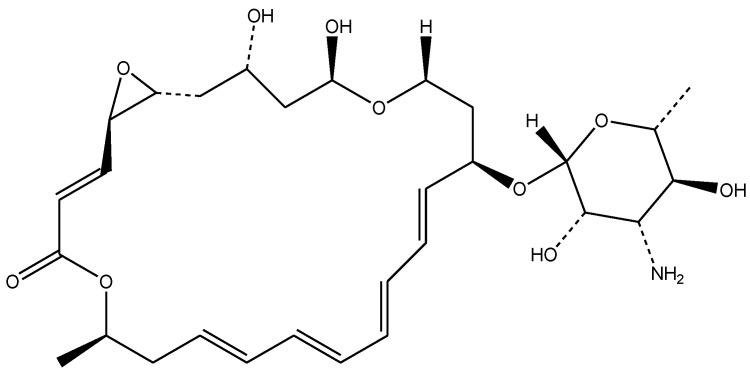
Structural formula of natamycin.

**Figure 10:**
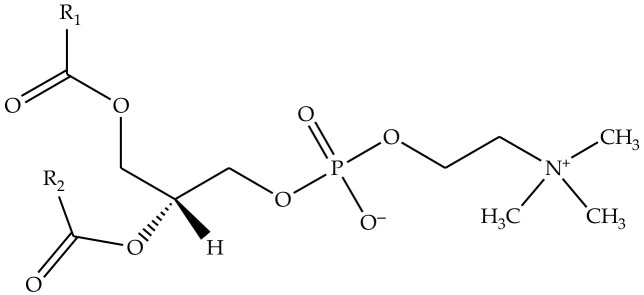
Structural formula of phosphatidylcholine (R_1_, and R_2_ represent acyl carbon chains).

The antifungal agent, natamycin Tredemix^®^ (Natamycin 50%), shown in Figure 9, was bought from Biokom Trendafilov Company, Belgrade, Serbia. The phospholipid mixtures, Phospholipon^®^ 90H (a commercial lipid mixture, containing hydrogenated phospholipids, with a content of ≥90.0%) and Lipoid^®^ S100 (a commercial lipid mixture, with a soybean phosphatidylcholine content of ≥94.0%), shown in Figure 10, as well as cholesterol (≥99%), were purchased from Lipoid Company, GmbH, Ludwigschafen, Germany. The used solvents, i.e., ethanol (96%, *v*/*v*), methanol, and chloroform, were obtained from Zorka Pharma, Šabac, Serbia. Triptic soy broth (TSB) with 2% glucose was from Torlak (Belgrade, Serbia). Yeast extract–Peptone–Dextrose (YPD), p-iodonitrotetrazolium violet, sodium phosphate monobasic (≥99%), and sodium phosphate dibasic dihydrate (≥98%) (used for the preparation of phosphate-buffered solution—PBS) were purchased from Sigma Aldrich, Schnelldorf, Germany. The MTT reagent (thiazolyl blue tetrazolium bromide, 1 mg/mL) was purchased from Sigma-Aldrich (St. Louis, MO, USA). The DMEM/F12 cell culture medium (1:1 mixture of Dulbecco’s Modified Eagle’s Medium and Ham’s F-12 nutrient mixture) was from Pan-Biotech (Aidenbach, Germany). Paraformaldehyde and glutaraldehyde were purchased from Sigma Aldrich (Steinheim, Germany). Water was purified using a Simplicity UV^®^ water purification system (Merck Millipore, Merck KGaA, Darmstadt, Germany). Crystal violet was from Bio-Mérieux (Marcy-l’Étoile, France), while sodium dodecyl sulfate (SDS) was from Sigma-Aldrich (Darmstadt, Germany). Spontaneously immortalized human keratinocytes (HaCaT cells) were from the Institute for Biological Research “Siniša Stanković”, National Institute of the Republic of Serbia, University of Belgrade, Belgrade, Serbia.

### 3.2. Preparation of Liposomal Vesicles via the Proliposome Technique

**Figure 11:**
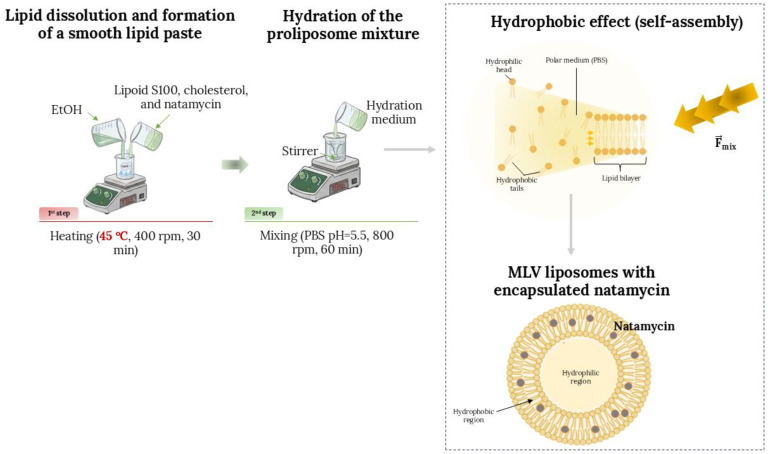
Schematic representation of liposome preparation using the proliposome method.

Cholesterol–phospholipid liposomal formulations containing the antifungal agent, natamycin, were prepared utilizing the proliposome method [20]. The liposomal formulations were prepared using two different commercial phospholipid mixtures, i.e., Lipoid S100 and Phospholipon 90H, as well as cholesterol. In short, 0.9 g of Lipoid S100 or Phospholipon 90H and cholesterol (0.125 g), and natamycin (30 mg) were mixed with 1.5 mL of ethanol and stirred at 45 °C, at a rotation speed of 400 rpm, for ~30 min, to allow for the solvent to completely evaporate and obtain a homogenous mixture. The used temperature is adequate, as it is sufficient for the removal of the solvent in its entirety, while it does not risk the degradation of the phospholipid. After the solvent removal, the aqueous phase (30 mL of PBS, pH ~ 5.5) was added in small portions, in order to provide the controlled hydration of the lipid film and to facilitate a gradual formation of liposomal particles. After the addition of the hydration medium was finished, the dispersion was stirred at 800 rpm for 1 h at room temperature. Plain liposomal vesicles (without the antifungal agent) were prepared as a control by leaving out the step of the addition of natamycin in the initial mixture. The entire process is depicted in Figure 11. The liposomal vesicles were kept at 4 °C, in the dark, until further analyses were performed.

### 3.3. Preparation of Liposomal Vesicles via the Thin Film Technique

**Figure 12:**
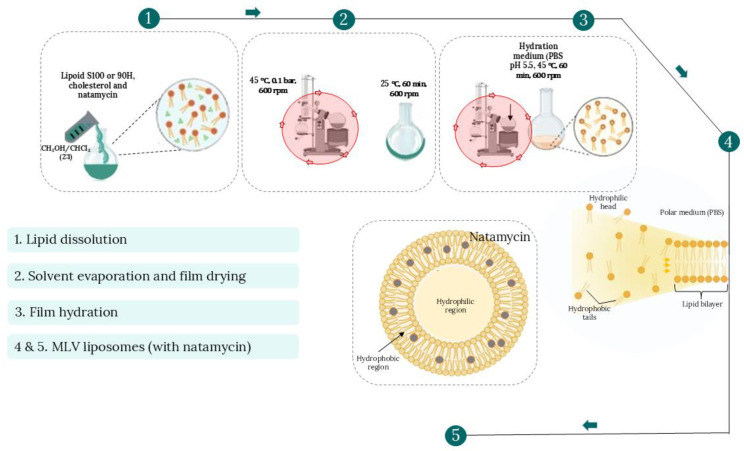
Schematic representation of liposome preparation using the thin film method.

Cholesterol–phospholipid liposomal particles containing natamycin were also prepared by the thin film method [11,41,75]. In essence, 1 g of the phospholipid mixture (Lipoid S100 or Phospholipon 90H) was combined with 30 mg of natamycin, as well as 0.150 mg of cholesterol, followed by the mixture’s dissolution in 5 mL of a 2:1 mixture of chloroform and methanol. After a clear, homogeneous mixture was obtained, the solvents were completely evaporated under a vacuum of 100 mm Hg using a rotary vacuum evaporator (IKA^®^-WERKE, HB4 basic, IKA, Staufen, Germany) at 45 °C (60 °C for Phospholipon 90H) and a rotation rate of 600 rpm to form a thin film. Evaporation continued for approximately 1 h until a dry residue was formed. By this method, the organic solvent is eliminated slowly to yield a thin lipid film on the interior surface of the flask, without causing thermal degradation of the phospholipid mixtures or cholesterol. The film was then hydrated with 30 mL of PBS with a pH of ~5.5 for 1 h at 45 °C (60 °C in case of Phospholipon 90H). As a control, plain liposomal vesicles (without natamycin) were also made using the same protocol. The entire process is depicted in Figure 12. All of the prepared liposomal formulations were kept at 4 °C in the dark until further analyses were performed.

### 3.4. Determination of the Encapsulation Efficiency

The natamycin-loaded liposomal particles were separated from the non-encapsulated fraction *via* centrifugation at 17,500 rpm and 4 °C, in two cycles lasting 45 min (Centrifuge 5430R, Eppendorf, Hamburg, Germany). The encapsulation efficiency (EE%) was determined by measuring the concentration of antifungal agent present in the supernatant after centrifugation, using BioTek Epoch 2 Elisa Microplate Spectrophotometer (Agilent Technologies, Santa Clara, CA, USA) at a wavelength of 304 nm (the wavelength at which the maximum absorption of natamycin is detected). The quantification of natamycin was performed using UV–Vis spectrophotometry by measuring the absorbance at 304 nm. Natamycin exhibits characteristic UV absorption peaks at approximately 291, 304, and 319 nm, with 304 nm commonly used for quantitative analysis [76]. Possible interference from liposomal components (phospholipids and sterol) was minimized by measuring the supernatant after centrifugation, where lipid content is expected to be negligible. Namely, the absorption of supernatant from blank liposomes (without natamycin) was also measured at 304 nm and showed negligible absorbance, confirming the specificity of the method.

EE% was calculated according to the concentration of natamycin present in the supernatant, as shown in Equation (1):

(1) $$EE\left[\%\right]=\frac{C_{i}-C_{sup}}{C_{i}}\times 100\%$$

C_i_ represents the initial concentration of the natamycin in the liposomal formulation, whereas C_sup_ stands for the natamycin concentration measured in the supernatant after centrifugation. Natamycin (0.02–1 mg/mL) was used as a standard to construct the calibration curve (equation: y = 18.3x + 1.071; linear regression, *R*^2^ = 0.9931). A good linearity was obtained (*R*^2^ = 0.9931), confirming the suitability of the method within the tested concentration range. The results for the EE% assessment were expressed as a percentage.

### 3.5. Transmission Electron Microscopy (TEM)

The morphology of natamycin-loaded liposomal vesicles was visualized by transmission electron microscopy (TEM). On formvar-coated 200-mesh copper grids, 10 µL of each liposome preparation was applied and allowed to adsorb at ambient temperature for 30 min. The grids were subsequently fixed with 2% paraformaldehyde for 10 min, rinsed three times with ultrapure water, and post-fixed with 2.5% glutaraldehyde for 5 min. Following a final rinse, the samples were air-dried and examined under a Philips CM12 transmission electron microscope (Philips, Eindhoven, The Netherlands).

### 3.6. Nanoparticle Tracking Analysis (NTA)

The particle concentration and size distribution of plain and natamycin-loaded liposomal vesicles, prepared using Phospholipon 90H or Lipoid S100 phospholipids and thin film or proliposome methods, were determined using a ZetaView^®^ QUATT PMX-430 nanoparticle tracking analyzer (Particle Metrix, Inning am Ammersee, Germany) operated with ZetaView software (version 8.05.16 SP3). Before analysis, the instrument was calibrated using 100 nm polystyrene beads. Liposomal suspensions were appropriately diluted with ultrapure water and analyzed in light-scattering mode, using a 488 nm blue laser. For each preparation, different dilution factors were used in order to obtain an optimal number of particles per frame (130–180 particles per frame), according to the manufacturer’s guidance. Measurement parameters included a shutter speed of 130, a frame rate of 30 frames per second, and a sensitivity level of 78. Data acquisition was performed with post-processing settings defined as a minimum area of 10, a maximum area of 1000, and a minimum brightness of 30. Each formulation was examined in triplicate at up to eleven positions, with meticulous cleaning between consecutive runs to prevent cross-contamination.

### 3.7. Determination of Zeta Potential and Polydispersity Index

The zeta potential and PDI of the developed liposomes (plain and natamycin-loaded) were evaluated using PCS. Measurements were carried out with a Zetasizer Nano ZS (Nano Series, Malvern Instruments Ltd., Malvern, UK), which is suitable for particle size analysis within the range of 0.6 nm to 6 µm. Before analysis, each sample was diluted 500 times with ultrapure water, and measurements were performed at 25 °C. All samples were analyzed in triplicate, and the results are reported as average values.

### 3.8. Determination of Density, Surface Tension, and Viscosity

The density and surface tension of developed liposomal formulations (empty and natamycin-loaded liposomal vesicles) were measured using a Force Tensiometer K20 (KRÜSS, Hamburg, Germany). Density was determined employing a silicon crystal as the immersion body, while surface tension was assessed using the Wilhelmy plate method. For each formulation, 20 mL of sample was analysed in triplicate at 25 °C. The results are presented as mean ± standard deviation and expressed in g/mL for density and mN/m for surface tension.

Viscosity measurements of the same liposomal samples were conducted using a Rotavisc *lo-vi* viscometer equipped with a VOL-C-RTD chamber, VOLS-1 adapter, and appropriate spindle (IKA, Staufen, Germany). A volume of 6.7 mL per sample was used, and each measurement was performed in triplicate at 25 °C. The data are presented as mean ± standard deviation and expressed in mPa·s.

### 3.9. Controlled Release Study

The *in vitro* comparative study of the controlled release of the antifungal drug, natamycin, from natamycin-loaded liposomal vesicles, as well as its solution containing the same concentration of the drug as in the liposomal vesicles, was performed using the Franz diffusion cell (PermGear, Inc., Hellertown, PA, USA). The donor and acceptor compartments of a Franz cell are divided by an acetate–cellulose membrane (Permgear, Hellertown, PA, USA) with a diffusion area of 4.91 cm^2^ and a pore size of 0.2 µm. The mentioned membrane served as an inert barrier to separate donor and receptor compartments and to evaluate drug release from the liposomal system under controlled conditions, rather than to simulate skin permeation. The donor compartment (0.05 g, d = 2.5 cm) contained the samples (2 mL of liposomal samples with natamycin, i.e., lower than 2 mg of natamycin due to the absence of the 100% of the encapsulation efficiency). A magnetic stirrer was used to continuously mix the release media (PBS, pH ~ 5.5, c = 0.1 mol/L) at 37 °C and 400 rpm in the receptor compartment [20,77]. For the duration of 24 h, samples were collected at set times (at 16 precisely determined time periods). All samples taken from the receptor compartment were replaced with 0.3 mL of fresh receptor medium, which was thermostated at 37 °C, to maintain synchronized operational parameters. Natamycin concentration was calculated for these samples after the absorbance measurements at 304 nm, using the calibration curve for natamycin mentioned in Section 2.4. *Determination of the Encapsulation Efficiency.* The results of each analysis were statistically processed after being run three times.

The release profiles were further analyzed to determine the diffusion coefficients and overall mass transfer resistance for natamycin released from the liposomal vesicles. The diffusion coefficients were calculated according to the method described by Pjanović et al. [78], based on Fick’s second law, as shown in Equation (2):

(2) $$\ln\left[\frac{C_{D}^{0}-C_{R}^{0}}{C_{D}-C_{R}}\right]=D\times \beta \times t$$

where C_D_ and C_R_ represent the concentrations of natamycin in the donor and receptor compartments, respectively, at time t, while $C_{D}^{0}$ and $C_{R}^{0}$ are the corresponding concentrations at t = 0. D is the diffusion coefficient, and β (2.49 × 10^4^ m^−2^) is a geometrical constant for a standard Franz diffusion cell (20 mL) [78]. The total mass transfer resistance (R) was calculated using the following Equation (3):

(3) $$R=\frac{\delta }{D}$$

In this equation, δ refers to the effective membrane thickness, which includes both the acetate–cellulose membrane and the layer (height) of liposomal vesicles, while D is the diffusion coefficient obtained from Equation (2). The value of R thus represents the combined resistance of the acetate–cellulose membrane and the liposomal vesicles.

The release of natamycin from the liposomal vesicles was compared to the release of pure natamycin from the solution that contained the same concentration of natamycin as the liposomal vesicles (30 mg in 30 mL of PBS), in order to determine the diffusion profile. The reference solution was prepared by dissolving the same amount of natamycin (30 mg) in the same amount of PBS used for the hydration step for liposomal preparation (30 mL). An ultrasound bath was used to aid the dissolution of natamycin.

The mechanisms governing the release of natamycin from the different liposome formulations were studied using three kinetic models, such as first-order kinetics (Equation (4)), Higuchi (Equation (5)), and Korsmeyer-Peppas (Equation (6)) models:

(4) $$\frac{m_{t}}{m_{\infty }}=100\cdot \left[1-e^{-kt}\right]$$

(5) $$\frac{m_{t}}{m_{\infty }}=k\cdot t^{1/2}$$

(6) $$\frac{m_{t}}{m_{\infty }}=k\cdot t^{n}$$

where m_t_ and m_∞_ are the amounts of released natamycin at time t and at infinite time, while k and t correspond to the release constant and release time, respectively. Exponent n determines the mechanism of natamycin release from the liposomal vesicles: 0.45 < n < 0.89 defines non–Fickian (anomalous) diffusion, n ≥ 0.89 defines an erosion mechanism, while n ≤ 0.45 defines Fickian diffusion [60,78,79,80,81].

### 3.10. Biological Activities of Natamycin-Loaded Liposomal Vesicles

#### 3.10.1. Measurement of the Antifungal Activity of Natamycin-Loaded Liposomal Vesicles

The antifungal potential of developed liposomal vesicles containing natamycin was tested against four *Candida* strains from the American Type Culture Collection (ATCC). For the bioassays, *Candida albicans* (ATCC 10231), *Candida lusitaniae* (ATCC 34449), *Candida auris* (CDC B 11903), and *Candida krusei* (ATCC 14243) were used. All fungal strains used in the test were from the Laboratory of Mycology, Department of Plant Physiology, University of Belgrade, Institute for Biological Research “Siniša Stanković”, National Institute of the Republic of Serbia, Belgrade, Serbia. In order to assess the antifungal activity of the natamycin-loaded liposomal vesicles, a modified version of the EUCAST procedure was employed [82]. Fungal species were cultured for 24 h at 37 °C in a YPD medium. The fresh *Candida* suspension was adjusted with YPD to a final concentration of 1.0 × 10^8^ per well. All of the tested natamycin-loaded liposomal vesicles had a concentration of the antifungal drug of 1 mg/mL. Minimal inhibitory concentration (MIC) and minimal fungicidal concentration (MFC) assessments were performed by a serial dilution method (the used concentrations were 20, 10, 5, 2.5, 1.5, 0.625, 0.3125, and 0.15625 mg/mL) in 96-well microtiter polystyrene plates. The MIC is defined as the lowest concentration of an antimicrobial agent that leads to the inhibition of visible growth of a microorganism under standardized *in vitro* conditions, i.e., the lowest sample concentration that reduced the color intensity in the wells (light red compared to the deep red in untreated controls) or led to a complete absence of color [83]. The MFC values were the lowest concentrations that eradicated fungal growth, indicating a 99.5% reduction in the initial inoculum. MIC and MFC values were determined by serial dilution of natamycin-loaded liposomal vesicles into microtiter plates containing broth (the wells contained 160 μL of liposomal suspension and 40 μL of medium). Then, the *Candida* suspension (10 μL) was added to each well, followed by a 24 h incubation at 37 °C. After incubation, *p*-iodonitrotetrazolium violet (0.04 mL, 0.2 mg/mL) was introduced as the coloring agent, and the plates were incubated at 37 °C for an additional 30 min. A natamycin solution of the identical concentration of natamycin as in the liposomal vesicles was used to assess the susceptibility of the tested strains to the pure antifungal agent, while blank liposomal vesicles were used as negative controls. The MIC and MBC values were provided in milligrams per milliliter.

#### 3.10.2. Crystal Violet Antibiofilm Assay

The antibiofilm activity was evaluated using the crystal violet (CV) assay previously reported in the literature according to Carević et al. [84], which quantifies the total biomass of adhered biofilms and is widely applied as a rapid initial screening method for assessing the antibiofilm potential of antimicrobial formulations. The effect of the tested natamycin solution, as well as natamycin-loaded and blank liposomal vesicles, on the ability of *C. krusei* and *C. lusitaniae* to form biofilms was evaluated. Fungal suspensions were exposed to each tested sample at concentrations corresponding to their MIC, 1/2 MIC, 1/4 MIC, and 1/8 MIC values. The assay was performed in 96-well microtiter plates with an adhesive surface (Sarstedt, Nümbrecht, Germany) using TSB supplemented with 2% glucose. Plates were incubated at 37 °C for 24 h. After incubation, non-adherent cells were removed by rinsing the wells twice with sterile PBS (100 µL, pH ~ 5.5), and the samples were air-dried. Methanol (100 µL) was then added for 10 min to fix the remaining attached cells. Once fixation was completed, the methanol was discarded, and the plates were left to dry at room temperature. Biofilms were stained with a 0.1% crystal violet solution (100 µL, Bio-Merieux, Craponne, France) for 30 min, followed by washing with distilled water and air-drying. The retained stain was solubilized using 96% ethanol, and absorbance was recorded with a Multiskan™ FC Microplate Photometer (Thermo Scientific™, Waltham, MA, USA) at 620 nm. The reduction in biofilm formation was calculated according to Equation (7):

(7) $$Biofilm\;destruction\left(\%\right)=\frac{A_{620,control}-A_{620,sample}}{A_{620,control}}\times 100\%$$

where A_620_, control corresponds to the absorbance of the untreated biofilm and A_620_, sample corresponds to the absorbance of the sample treated with the tested formulation.

#### 3.10.3. Cytotoxicity Analysis

HaCaT cells were cultured in DMEM-F12 medium supplemented with 10% FBS and 1% antibiotic/antimycotic solution and seeded into 96-well plates at a density of 1.5 × 10^4^ cells per well, with a final volume of 100 µL in each well. After 24 h of incubation, the culture medium was replaced, and the cells were exposed to the treatments (natamycin solution, plain or natamycin-loaded liposomal vesicles) in a range of concentrations (1, 10, and 100 µg/mL of natamycin, i.e., 0.1, 1, and 10% of liposomal vesicles) or the negative control in a total volume of 100 µL per well. Following 24 h of incubation at 37 °C, cell viability was evaluated using the MTT assay. For this purpose, 10 µL of MTT reagent was added to each well, and the plates were incubated for 2 h at 37 °C in the dark to allow the formation of formazan crystals. The resulting crystals were solubilized using an SDS solution [85]. Absorbance was then recorded at 570 nm using a microplate reader (Epoch, BioTek, Shoreline, WA, USA) after complete dissolution of the crystals. Cell viability was expressed as a percentage relative to the untreated control (set as 100%). The data represent mean values from three independent experiments performed in triplicate (n = 9).

### 3.11. Statistical Analysis

The statistical analysis was performed using analysis of variance (one-way ANOVA) and Duncan’s *post hoc* test within the software STATISTICA 7.0 (TIBCO Software Inc., Palo Alto, CA, USA). The differences were deemed statistically significant at *p* < 0.05, n = 3. For the cell-based assay, as well as encapsulation efficiency, data were first tested for normality, and differences between treatments and controls were analyzed using one-way analysis of variance (ANOVA) followed by Tukey’s *post hoc* test. Results are presented as means ± standard deviations (SDs). Statistical analyses were performed using GraphPad Prism 6.0 (GraphPad Software, Inc., La Jolla, CA, USA), and *p* < 0.05 was considered statistically significant (n = 9).

## 4. Conclusions

This study aimed to evaluate the influence of different preparation methods and phospholipid compositions on the physicochemical properties, biological activity, and safety profile of natamycin-loaded liposomal vesicles as a preliminary step toward their potential use in topical delivery systems. When subjected to the assessment of EE%, all of the prepared liposomal formulations had a high EE%, with the highest in the case of liposomal vesicles developed using Lipoid S100 and the proliposome method. TEM analysis confirmed that the preparation method plays a decisive role in determining liposome morphology and structural integrity, with the thin-film hydration technique producing more uniform and well-defined vesicles compared to the proliposome method. The incorporation of natamycin resulted in reduced vesicle size and increased particle concentration (determined in the NTA), confirming favorable interactions between the drug and phospholipid bilayers, particularly in the case of the proliposome method. The differences in vesicle size and particle concentration between Lipoid S100 and Phospholipon 90H liposomal vesicles confirm that phospholipid composition, particularly the degree of acyl chain saturation and bilayer fluidity, critically determines liposome structure, liposome stability, and formation efficiency. The physical analysis demonstrated that all liposomal systems exhibited comparable density, confirming their predominantly aqueous composition and structural stability. However, variations in surface tension and viscosity reflected the influence of phospholipid type, preparation technique, and natamycin incorporation on the interfacial and rheological behavior of the dispersions. Namely, the proliposome-derived and drug-loaded liposomal vesicles showed slightly improved formulation uniformity according to the NTA measurements. The PCS results confirm that lipid composition and preparation technique play a crucial role in determining liposome uniformity and stability, with 90H-based thin film formulations demonstrating the most favorable zeta potential value. The *in vitro* release-based comparative study showed that encapsulation of natamycin into liposomal vesicles leads to a prolonged *in vitro* release of the antifungal agent. The liposomal formulation obtained by employing Lipoid S100 and the proliposome technique had the slowest release rate. The antifungal and antibiofilm assays showed that liposomal vesicles prepared using the Phospholipon 90H lipid mixture have a higher ability to inhibit fungal growth, as well as biofilm formation. The cytotoxicity of natamycin-loaded liposomal vesicles was concentration-dependent, with all formulations maintaining cell viability above 80% even at the highest tested dose, highlighting an acceptable balance between antifungal efficacy and cellular compatibility. The slightly higher cytotoxicity of Phospholipon 90H/proliposome-based liposomal vesicles compared to Lipoid S100/thin film liposomal vesicles is likely due to the phospholipid composition, as Phospholipon 90H may interact more strongly with cell membranes than Lipoid S100. At lower therapeutic concentrations, the formulations were biocompatible and, in some cases, slightly enhanced keratinocyte metabolic activity, suggesting favorable *in vitro* cytocompatibility for further investigation in topical delivery applications. All of the results obtained in this study show that the method used for liposomal preparation did not have a strong influence on the tested parameters, but rather the lipid mixture that was used.

This study presents an initial comparative evaluation of two liposome preparation methods and two lipid mixtures, aiming to identify the optimal formulation for natamycin encapsulation and *in vitro* characterization for potential application in topical delivery systems. Even though this research highlights the potential advantages of liposomal carriers for topical delivery systems, further studies are required to evaluate aspects such as sensory properties, scale-up feasibility, and other formulation-related parameters. Future research should focus on additional *in vivo* studies and further evaluation of the developed liposomal vesicles for their potential incorporation into topical delivery systems. In future studies, Cryo-TEM characterization should be performed to enable visualization of liposomal vesicles under near-native conditions and further elucidate vesicle morphology and the spatial distribution of natamycin within the liposomal system. Additionally, *ex vivo* skin permeation and retention experiments should be conducted to comprehensively evaluate liposome delivery potential for topical natamycin, as well as leakage study and temperature-dependent stability experiments to fully evaluate the structural integrity and performance of the liposomal formulations.

## Figures and Tables

**Table 1:** Density, surface tension, and viscosity of plain and natamycin-loaded liposomal vesicles prepared using Phospholipon 90H or Lipoid S100 phospholipids in thin film or proliposome methods.

Samples			Density (g/mL)	Surface Tension (mN/m)	Viscosity (mPa·s)
Thin film method	90H	Plain liposomal vesicles	0.999 ± 0.001 ^b^ *	26.67 ± 1.47 ^ab^	1.65 ± 0.03 ^e^
		Liposomal vesicles with natamycin	1.000 ± 0.002 ^b^	28.00 ± 1.40 ^a^	1.88 ± 0.05 ^bc^
	S100	Plain liposomal vesicles	1.007 ± 0.001 ^a^	22.63 ± 0.91 ^cd^	1.77 ± 0.04 ^de^
		Liposomal vesicles with natamycin	1.007 ± 0.001 ^a^	24.32 ± 1.16 ^bc^	1.89 ± 0.04 ^bc^
Proliposome method	90H	Plain liposomal vesicles	0.997 ± 0.002 ^b^	21.67 ± 0.61 ^d^	1.76 ± 0.01 ^e^
		Liposomal vesicles with natamycin	1.001 ± 0.002 ^b^	23.20 ± 1.08 ^cd^	2.26 ± 0.20 ^a^
	S100	Plain liposomal vesicles	1.006 ± 0.001 ^a^	22.60 ± 0.40 ^d^	1.82 ± 0.03 ^cd^
		Liposomal vesicles with natamycin	1.007 ± 0.001 ^a^	22.87 ± 0.12 ^d^	1.96 ± 0.05 ^b^

* Different superscript letters represent statistically significant differences between samples for each variable separately (n = 3, one-way analysis of variance and Duncan’s multiple range test).

**Table 2:** Diffusion coefficients (D) and diffusion resistance (R) of natamycin (N) solution and natamycin-loaded liposomal vesicles prepared by thin-film (tf) and proliposome (pl) methods using Lipoid S100 and Phospholipon 90H phospholipids at pH ~ 5.5.

Sample	δ, m	D, m^2^/s	R, s/m	R_LIP_, s/m
S100 + N, tf	4.07 × 10^−3^	1.38 × 10^−10^	2.96 × 10^7^	2.957 × 10^7^
90H + N, tf		3.01 × 10^−10^	1.35 ×10^7^	1.351 × 10^7^
S100 + N, pl		1.37 × 10^−10^	2.97 × 10^7^	2.966 × 10^7^
90H + N, pl		1.79 × 10^−10^	2.27 × 10^7^	2.266 × 10^7^
Natamycin solution		1.93 × 10^−7^	2.11 × 10^4^	n.a. *

* not applicable.

## Data Availability

The original contributions presented in this study are included in the article/Supplementary Materials. Further inquiries can be directed to the corresponding author.

## Supplementary Materials

The following supporting information can be downloaded at: https://www.mdpi.com/article/10.3390/ph19050710/s1, Figure S1: Representative transmission electron microscopy images of natamycin-loaded liposomal vesicles: (A) 90H + nat, proliposome, (B) S100 + nat, proliposome, and (C) S100+ nat, thin film; bar—2 µm. The arrow points to the natamycin particles; whereas the arrowhead shows the liposomal vesicle. Figure S2: Kinetic fitting of natamycin release from the natamycin solution, using the Higuchi, First-order, and Korsmeyer-Peppas models. Fitting data are represented by lines, while symbols refer to experimental data of cumulative natamycin release from liposomes. Figure S3: Kinetic fitting of natamycin release from the tested liposomal formulations prepared by the proliposome method and Lipoid S100 phospholipid, using the Higuchi, First-order, and Korsmeyer-Peppas models. Fitting data are represented by lines, while symbols refer to experimental data of cumulative natamycin release from liposomes. Figure S4: Kinetic fitting of natamycin release from the tested liposomal formulations prepared by the thin-film method and Lipoid S100 phospholipid, using the Higuchi, First-order, and Korsmeyer-Peppas models. Fitting data are represented by lines, while symbols refer to experimental data of cumulative natamycin release from liposomes. Figure S5: Kinetic fitting of natamycin release from the tested liposomal formulations prepared by the proliposome method and Phospholipon 90H phospholipid, using the Higuchi, First-order, and Korsmeyer-Peppas models. Fitting data are represented by lines, while symbols refer to experimental data of cumulative natamycin release from liposomes. Figure S6: Kinetic fitting of natamycin release from the tested liposomal formulations prepared by the thin-film method and Phospholipon 90H phospholipid, using the Higuchi, First-order, and Korsmeyer-Peppas models. Fitting data are represented by lines, while symbols refer to experimental data of cumulative natamycin release from liposomes. Table S1: Polydispersity index (PDI) and zeta potential of blank (control) and natamycin-loaded liposomal vesicles prepared using thin film and proliposome techniques, and Lipoid S100 and Phospholipon 90H phospholipids, determined using photon correlation spectroscopy. Table S2: Model parameters for First-order kinetics of natamycin release from liposomes and natamycin solution into pH = 5.5 phosphate-buffered saline medium. Table S3: Model parameters for Higuchi kinetics of natamycin release from liposomes and natamycin solution into pH = 5.5 phosphate-buffered saline medium. Table S4 Model parameters for Korsmeyer-Peppas kinetics of natamycin release from liposomes and natamycin solution into pH = 5.5 phosphate-buffered saline medium.

## Abbreviations

The following abbreviations are used in this manuscript:

PBS	Phosphate-Buffered Saline
TSB	Tryptic Soy Broth
EE	Encapsulation Efficiency
TEM	Transmission Electron Microscopy
NTA	Nanoparticle Tracking Analysis
ATCC	American Type Culture Collection
CDC	Disease Control and Prevention
HaCaT	Human keratinocytes
YPD	Yeast extract–Peptone–Dextrose
