# Supplementary material for: Development and Characterization of Natamycin-Loaded Liposomes for Potential Topical Application: Influence of Preparation Method and Phospholipid Composition
Source: Pharmaceuticals (Basel). 2026 Apr 30;19(5):710. doi: 10.3390/ph19050710 (PMC13209329; doi:10.3390/ph19050710)
Supplement: Supplementary file 1 [file pharmaceuticals-19-00710-s001.zip › pharmaceuticals-4268924-supplementary.pdf]

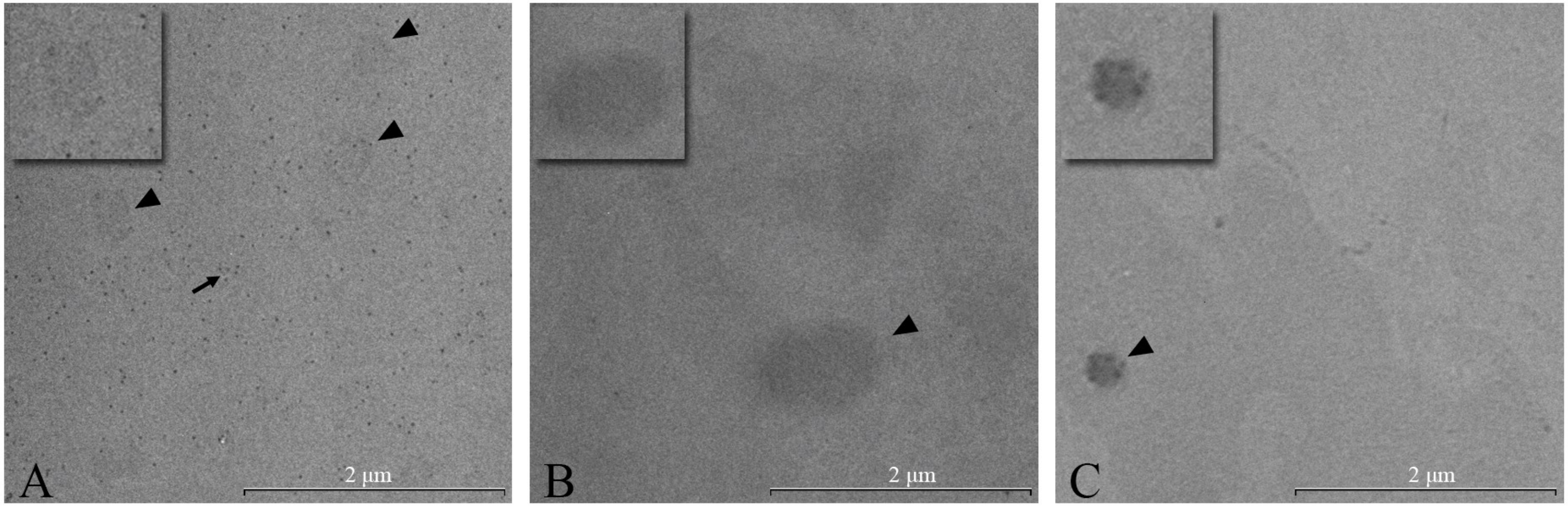


*Figure S 1 Representative transmission electron microscopy images of natamycin-loaded liposomal vesicles: (A) 90H + nat, proliposome, (B) S100 + nat, proliposome, and (C) S100+ nat, thin film; bar - 2 μm. The arrow points to the natamycin particles; whereas the arrowhead shows the liposomal vesicle.*

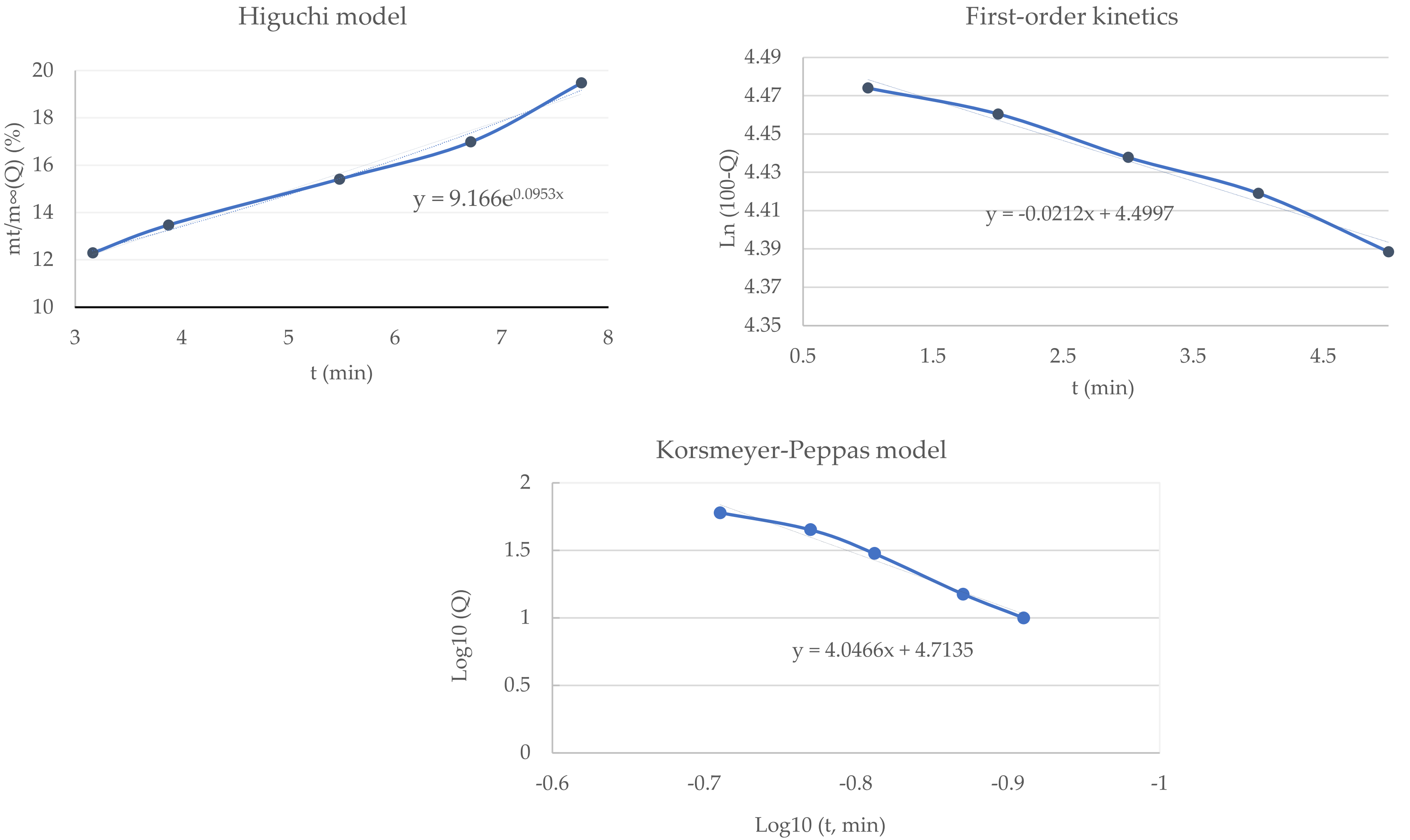


*Figure S2 Kinetic fitting of natamycin release from the natamycin solution, using the Higuchi, First-order, and Korsmeyer-Peppas models. Fitting data are represented by lines, while symbols refer to experimental data of cumulative natamycin release from liposomes.*

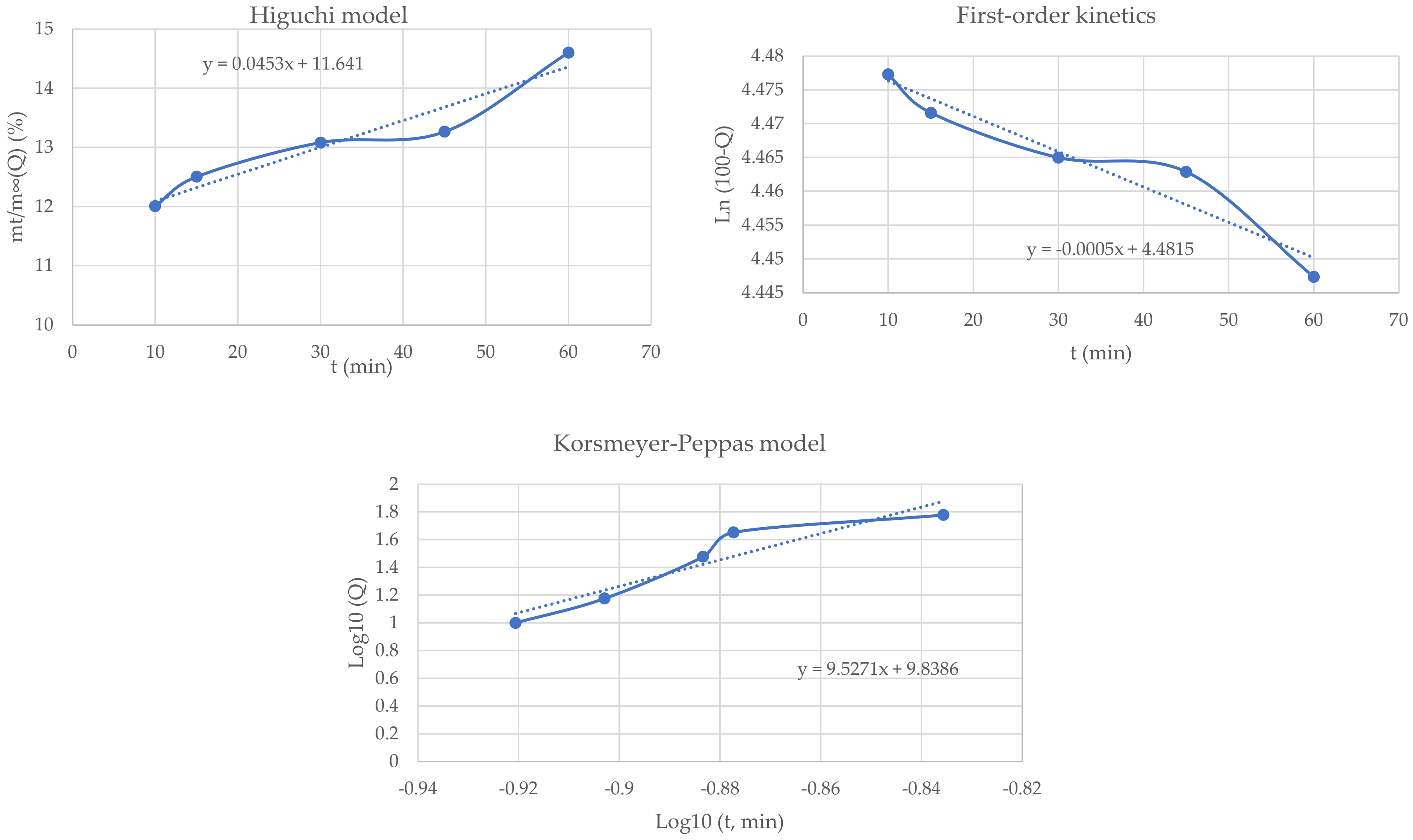


*Figure S 3 Kinetic fitting of natamycin release from the tested liposomal formulations prepared by the proliposome method and Lipoid S100 phospholipid, using the Higuchi, First-order, and Korsmeyer-Peppas models. Fitting data are represented by lines, while symbols refer to experimental data of cumulative natamycin release from liposomes.*

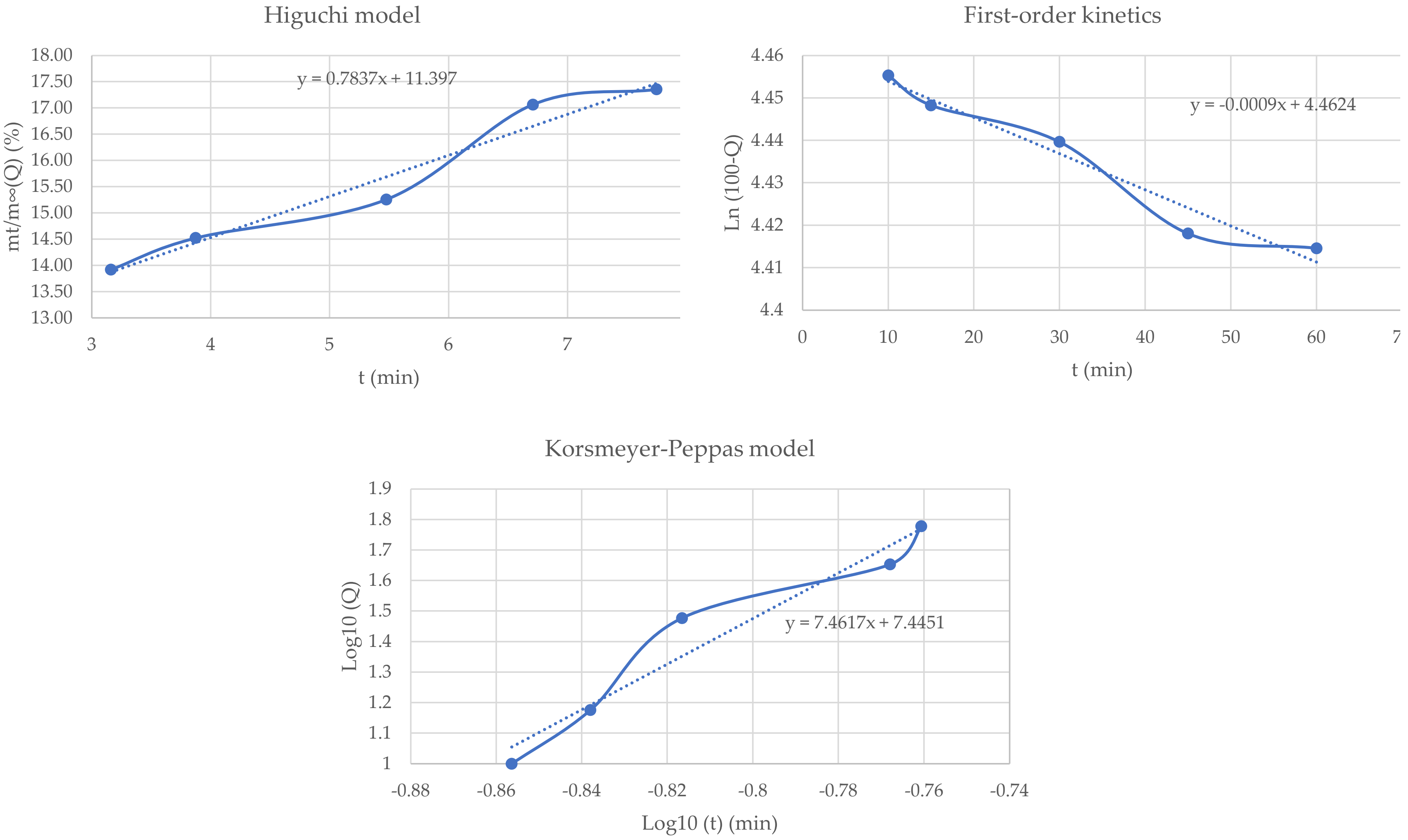


*Figure S 4 Kinetic fitting of natamycin release from the tested liposomal formulations prepared by the thin-film method and Lipoid S100 phospholipid, using the Higuchi, First-order, and Korsmeyer-Peppas models. Fitting data are represented by lines, while symbols refer to experimental data of cumulative natamycin release from liposomes.*

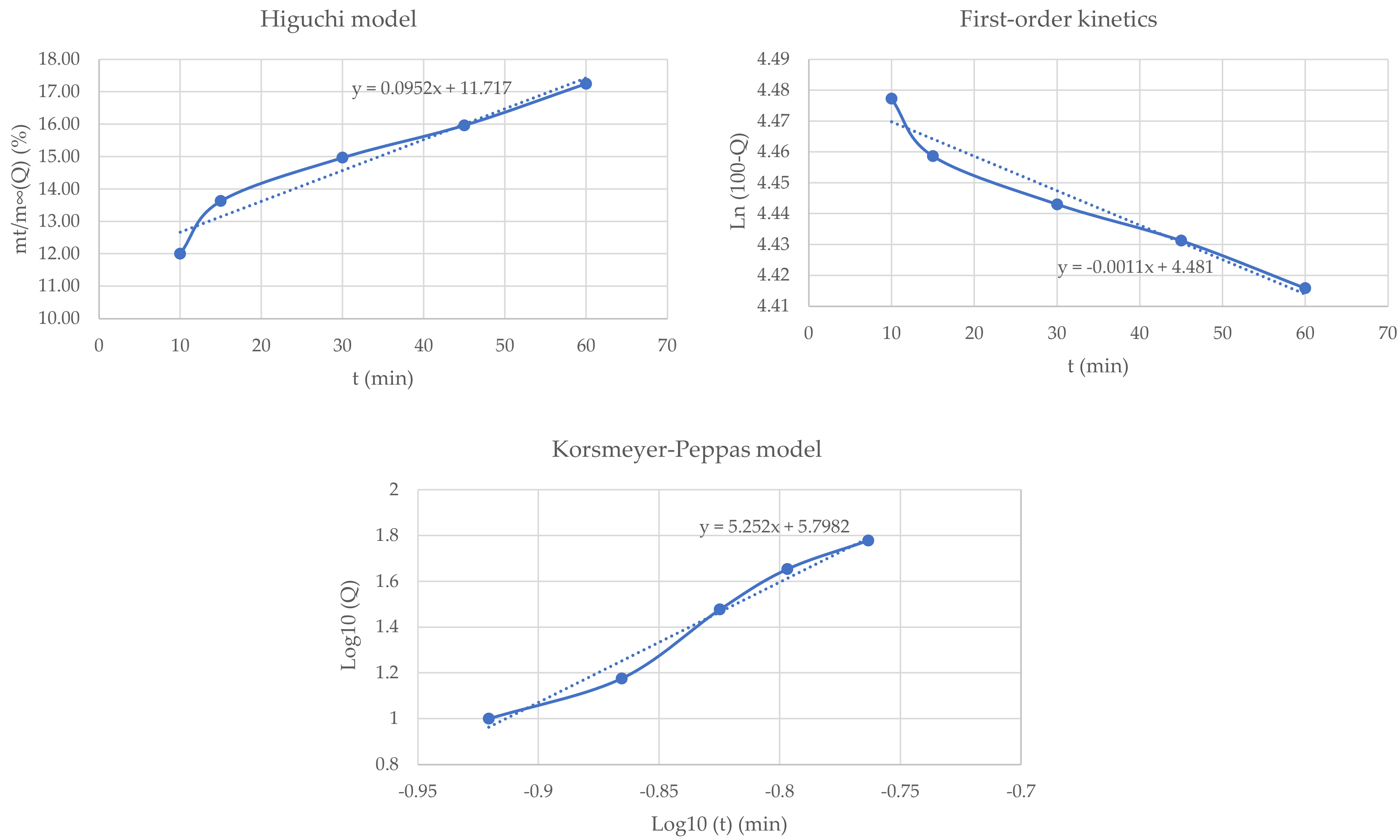


*Figure S 5 Kinetic fitting of natamycin release from the tested liposomal formulations prepared by the proliposome method and Phospholipon 90H phospholipid, using the Higuchi, First-order, and Korsmeyer-Peppas models. Fitting data are represented by lines, while symbols refer to experimental data of cumulative natamycin release from liposomes.*

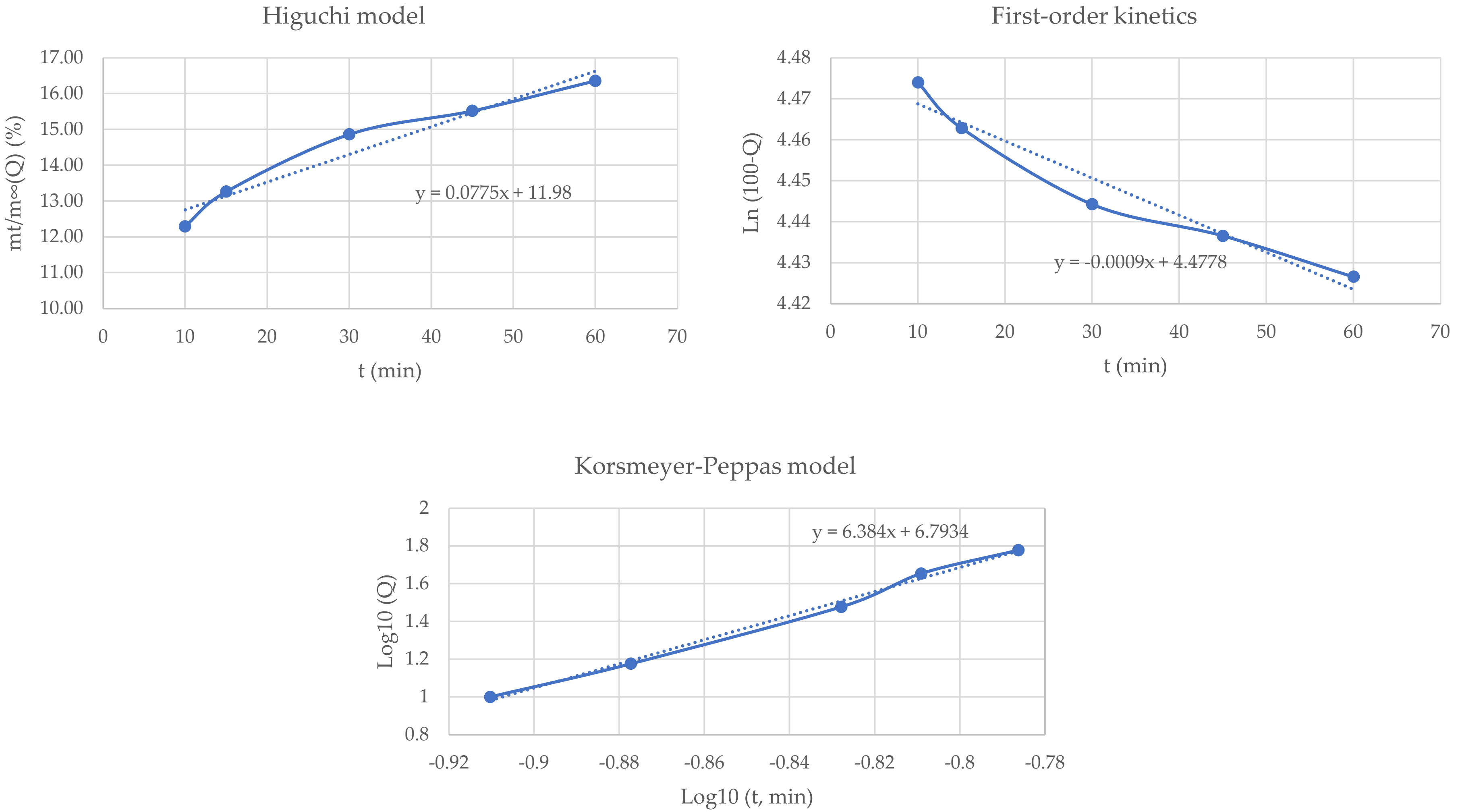


*Figure S 6 Kinetic fitting of natamycin release from the tested liposomal formulations prepared by the thin-film method and Phospholipon 90H phospholipid, using the Higuchi, First-order, and Korsmeyer-Peppas models. Fitting data are represented by lines, while symbols refer to experimental data of cumulative natamycin release from liposomes.*

**Table S1** Polydispersity index (PDI) and zeta potential of blank (control) and natamycin-loaded liposomal vesicles prepared using thin film and proliposome techniques, and Lipoid S100 and Phospholipon 90H phospholipids, determined using photon correlation spectroscopy

| Sample | PDI | Zeta potential (mV) |
|---|---|---|
| 90H + natamycin, thin film | 0.387±0.115 | -37.98±0.98 |
| 90H, thin film | 0.546±0.114 | -41.00±1.57 |
| S100 + natamycin, thin film | 0.321±0.112 | -32.90±2.07 |
| S100, thin film | 0.356±0.089 | -28.54±0.98 |
| 90H + natamycin, proliposome | 0.654±0.115 | -32.15±1.15 |
| 90H, proliposome | 0.721±0.118 | -31.24±2.20 |
| S100 + natamycin, proliposome | 0.367±0.067 | -30.23±2.56 |
| S100, proliposome | 0.836±0.110 | -35.32±1.59 |

**Table S2** Model parameters for First-order kinetics of natamycin release from liposomes and natamycin solution into pH=5.5 phosphate-buffered saline medium

| Formulation | Equation (First-order kinetics) | Parameter $R^2$ |
|---|---|---|
| 90H + natamycin, proliposome | $\frac{m_t}{m_\infty} = 100 \cdot [1 - e^{-kt}]$ | 0.951 |
| S100 + natamycin, thin film | | 0.955 |
| 90H + natamycin, thin film | | 0.947 |
| S100 + natamycin, proliposome | | 0.926 |
| Natamycin solution, 1 mg $mL^{-1}$ | | 0.976 |

**Table S3** Model parameters for Higuchi kinetics of natamycin release from liposomes and natamycin solution into pH=5.5 phosphate-buffered saline medium

| Formulation | Equation (Higuchi) | Parameter $R^2$ |
|---|---|---|
| 90H + natamycin, proliposome | $\frac{m_t}{m_\infty} = \mathrm{k} \cdot t^{1/2}$ | 0.983 |
| S100 + natamycin, thin film | | 0.944 |
| 90H + natamycin, thin film | | 0.947 |
| S100 + natamycin, proliposome | | 0.959 |
| Natamycin solution, 1 mg $mL^{-1}$ | | 0.927 |

**Table S4** Model parameters for Korsmeyer-Peppas kinetics of natamycin release from liposomes and natamycin solution into pH=5.5 phosphate-buffered saline medium

| Formulation | Equation (Korsmeyer-Peppas) | Parameter $R^2$ |
|---|---|---|
| 90H + natamycin, proliposome | $\frac{m_t}{m_\infty} = \mathrm{k} \cdot t^n$ | 0.978 |
| S100 + natamycin, thin film | | 0.946 |
| 90H + natamycin, thin film | | 0.995 |
| S100 + natamycin, proliposome | | 0.879 |
| Natamycin solution, 1 mg mL$^{-1}$ | | 0.976 |
